# Programmable dynamic steady states in ATP-driven nonequilibrium DNA systems

**DOI:** 10.1126/sciadv.aaw0590

**Published:** 2019-07-19

**Authors:** Laura Heinen, Andreas Walther

**Affiliations:** 1 Institute for Macromolecular Chemistry, University of Freiburg, Stefan-Meier-Straße 31, 79104 Freiburg, Germany.; 2 Freiburg Materials Research Center (FMF), University of Freiburg, Stefan-Meier-Straße 21, 79104 Freiburg, Germany.; 3 Freiburg Center for Interactive Materials and Bioinspired Technologies (FIT), University of Freiburg, Georges-Köhler-Allee 105, 79110 Freiburg, Germany.; 4 Freiburg Institute for Advanced Studies (FRIAS), University of Freiburg, Albertstraße 19, 79104 Freiburg, Germany.

## Abstract

Inspired by the dynamics of the dissipative self-assembly of microtubules, chemically fueled synthetic systems with transient lifetimes are emerging for nonequilibrium materials design. However, realizing programmable or even adaptive structural dynamics has proven challenging because it requires synchronization of energy uptake and dissipation events within true steady states, which remains difficult to orthogonally control in supramolecular systems. Here, we demonstrate full synchronization of both events by ATP-fueled activation and dynamization of covalent DNA bonds via an enzymatic reaction network of concurrent ligation and cleavage. Critically, the average bond ratio and the frequency of bond exchange are imprinted into the energy dissipation kinetics of the network and tunable through its constituents. We introduce temporally and structurally programmable dynamics by polymerization of transient, dynamic covalent DNA polymers with adaptive steady-state properties in dependence of ATP fuel and enzyme concentrations. This approach enables generic access to nonequilibrium soft matter systems with adaptive and programmable dynamics.

## Teaser

Chemical fuel and reaction networks program nonequilibrium steady-state structural dynamics in dynamic covalent DNA systems.

## INTRODUCTION

Biological systems operate out of equilibrium under constant influx of energy and matter, and are orchestrated via signaling and reaction networks (*1*–*3*). For example, microtubules and actin filaments polymerize dynamically by consumption of chemical fuels and persist in a fueled dynamic steady state (DySS) with unusual dynamics (e.g., instabilities) needed for rapid spatiotemporal reorganization in the cytoskeleton (*1*, *4*). Mimicking such biological dissipative structures with tunable structural dynamics in their steady states remains a profound challenge in the emergent pursuit for artificial, nonequilibrium molecular systems, but, at the same time, represents one of the most critical aspects for the design of next-generation autonomous, active matter–type, functional material systems with truly adaptive or even life-like properties (*5*–*8*).

Research on chemically fueled systems has so far mostly focused on supramolecular structures, in which monomeric building blocks are embedded into a kinetically controlled reaction network and therein temporarily activated for self-assembly (*9*–*12*). However, energy-driven structural dynamics in such systems—with simultaneous formation, collapse, and exchange of the structural units—is enabled only when chemical activation and deactivation occur concurrently and critically synchronize appropriately with the kinetics of structure formation and destruction. Fiber dynamics were first and only reported for Me_2_SO_4_-fueled supramolecular self-assemblies of carboxylate gelator molecules using transient esterification in alkaline hydrolytic environments (*13*), while it was not reported for other supramolecular fibrils of partly very similar structure (*14*–*17*). Structural dynamics are even harder to realize in fuel-dissipating environments with a modulated self-assembly trigger [e.g., pH or adenosine 5′-triphosphate (ATP)], because deactivation of the fueling signal occurs for kinetic reasons preferentially outside the structure (*18*–*27*). For instance, although highly valuable for designing autonomous systems with lifetimes, recent examples of ATP- or pH-triggered transient self-assemblies, which use enzymes to control the concentrations of such triggering signals, have an unfavorable kinetic situation to reach energy-driven dynamics in their transient states. This is simply due to the fact that free signaling molecules outside the assemblies are kinetically more accessible for conversion and degradation, leading to a situation where the assembly reacts as a whole to a changing environment (*18*–*27*).

Beyond such ATP-responsive self-assemblies with transient signal dissipation (*18*–*20*, *25*, *26*), ATP-fueled supramolecular peptide fibrils were reported by direct enzymatic phosphorylation of peptide residues and concurrent removal of it (*28*). In a dialysis reactor with continuous waste removal and fuel supply, steady states were successfully sustained; however, structural dynamics remain elusive as the fibrils undergo unfavorable higher-level aggregation.

Here, we step away from supramolecular structures and introduce the first example of a chemically fueled dissociative dynamic covalent bond system (*29*–*31*). Critically, this strategy enables facile access to adaptive and programmable structural DySSs by mechanistically synchronizing the energy events (uptake/dissipation) with structural transitions (bond formation/cleavage). In more detail, we present the ATP-fueled activation and dynamization of covalent phosphodiester DNA bonds via an enzymatic reaction network of concurrently acting ATP-dependent DNA ligase and counteracting endonuclease, which modulate jointly the average steady-state bond ratio and bond exchange frequencies. Bridging the fields of DNA nanotechnology and polymer science, we transduce this concept to nonequilibrium dynamic covalent and transient DNA chain growth with programmable DySS properties. The ATP fuel level in the system primarily programs the lifetime, whereas the kinetic balance between the ligation and the restriction reaction, as encoded by the concentrations and ratios of the enzymes, dictates the average steady-state chain length, dispersity, and the exchange frequencies of the polymer chains. Our approach introduces a generic dynamic covalent bond as a new concept into nonequilibrium DNA nanoscience (*23*, *32*–*37*). Moreover, we suggest chemically fueled dissipative dynamic covalent bonds as a generic concept for the nascent field of dissipative nonequilibrium systems design, which allows engineering of functional active matter with adaptive and autonomously programmable DySS behavior.

## RESULTS

### ATP-fueled dynamization of covalent DNA bonds

**Fig. 1:**
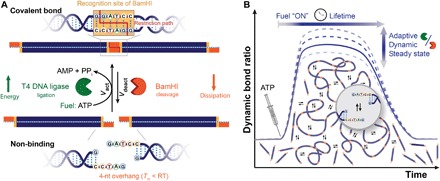
ATP-fueled dynamization of phosphodiester bonds by simultaneous action of two antagonistic DNA enzymes for transient dynamic covalent polymerization of DNA strands with tunable lifetimes and adjustable steady-state dynamics. (**A**) Short telechelic DNA monomers, M_1_, with 4-nt self-complementary ssDNA ends are covalently joined via T4 DNA ligase–catalyzed phosphodiester bond formation under consumption of two ATP fuel molecules. This ligation forms the recognition site (highlighted by the orange box) of the endonuclease BamHI, which counteracts ligation by catalyzing the cleavage (restriction path as red line) of the just formed phosphodiester bonds. Simultaneous ligation and cutting at this site creates a dynamic covalent bond until the ATP runs out. (**B**) Transient growth of dynamically polymerizing DNA chains in a closed system is achieved by a faster ligation than restriction reaction (*v*_act_ ≫ *v*_deact_). The lifetime is coupled to the ATP fuel and can be tuned together with the DySS properties of the dynamic covalent DNA chains under biocatalytic control.

Our concept enabling this first example of a chemically fueled dynamic covalent bond with direct implications for higher-level structural dynamics builds on the ATP-fueled enzymatic activation and dynamization of a DNA phosphodiester bond in the presence of antagonistic enzymes joining and cutting this linkage. We apply this concept directly to the transient dynamic chain growth polymerization of α,ω-telechelic DNA monomer strands, M_1_ (Fig. 1A). M_1_ is a rigid duplex of 34 base pairs (bp) with a self-complementary single-stranded DNA (ssDNA) 4-nucleotide (nt) overhang at each side. These ssDNA ends carry the molecular recognition information to self-extend, but are too short to stably connect M_1_ into elongated chains, as the 4-bp hybridization has a low melting temperature, *T*_m_ ≈ 0°C (fig. S1). However, joining of two ends can be achieved by T4 DNA ligase, which catalyzes the phosphodiester bond formation between adjacent 5′-phosphate and 3′-OH groups in a DNA duplex under consumption of one ATP molecule (fig. S1). Coupling of two M_1_ requires two ligation steps and consumes two molecules of ATP. The M_1_ ends are designed in a way that successful ligation creates the recognition site (GGATCC, orange box, Fig. 1A) for an antagonistic restriction enzyme, BamHI. BamHI cuts the double-stranded DNA (dsDNA) strands by hydrolytic cleavage of the phosphodiester bond at the position where the M_1_ strands were just ligated. Cleavage is thus conditional on previous ligation, and the phosphodiester bond formation is fully reversible. The ligation transfers chemical energy from ATP into a covalent phosphodiester bond in the DNA backbone, while the restriction enzyme dissipates this energy by breaking these bonds hydrolytically. The simultaneous action of both enzymes creates a dynamized phosphodiester bond under biocatalytic control. The kinetic boundary condition for the formation of a transient polymer state is that ligation is faster than cleavage. The overall lifetime is given by the availability and consumption of chemical fuel and the concentrations of the enzymes, whereas the enzyme concentrations modulate the reaction frequencies needed to program the dynamics of the transient DySS.

The reaction network embedding the ATP-fueled dynamic phosphodiester bond fulfills the relevant features for the formation of a dissipative nonequilibrium system: (i) Structure formation is coupled to an energy-fueled activation (ATP-dependent ligation). (ii) The deactivation dissipates energy [cleavage of a covalent bond, Δ*G* = −5.3 kcal/mol; (*38*)]. (iii) Activation and deactivation are chemically independent, selective, and kinetically tunable reactions. (iv) The structure is completely reversible on a molecular level. Consequently, this ATP-fueled dynamization of a phosphodiester bond constitutes a general strategy to establish dissipative DNA-based structures and energy-driven active materials.

Critically, the chemical fuel acts only as an energy-providing source (a cosubstrate) to form the bond and connect DNA strands of choice (fig. S1) but is not integrated into the structures as a terminal group. This is decisive to program larger molecular architectures and opens considerable flexibility for rational design of functionalities and connectivity patterns.

Moreover, the present dissipative system fully synchronizes energetic and structural events, which provides the key advantage to mechanistically embed structural dynamics in the DySS. It enables deterministic access to material properties such as tunable exchange frequencies important for self-repair and adaptation in fueled DySSs.

### Transient DySS DNA polymerization system with ATP-dependent lifetimes

Dynamic covalent polymerization of DNA chains requires four main components (Fig. 1B): the DNA monomer M_1_, T4 DNA ligase, BamHI restriction enzyme, and ATP as a chemical fuel in a suitable buffer. Without ATP, the system is inactive. After addition of ATP, DNA chains grow immediately and evolve into a DySS with continuous joining and cutting. Once the ATP level becomes subcritical, cutting events overpower ligation and the DNA chains degrade back to the initial state. Each of the four-system components and the reaction temperature control the dynamic polymerization and program its DySS properties as detailed below by systematic kinetic studies.

The basic reaction conditions were derived from extensive screening of the individual reactions as summarized in Supplementary Note A (figs. S2 to S5). The DNA concentration [M_1_] = 0.05 mM was set as a fixed parameter in all kinetic experiments. All experiments contain at least equimolar ATP (related to the number of possible ligation sites, i.e., [ATP] ≥ 2•[M_1_]) to avoid limitations in chain length from low conversion in the fueled step growth-like polymerization (figs. S3 and S9). From the individual kinetics of the enzyme-dependent DNA chain growth and degradation experiments (figs. S2 and S5), we found 41.25 WU (Weiss units; Supplementary Note A) of T4 DNA ligase and 900 U of BamHI as a suitable enzyme ratio fulfilling the kinetic requirement of a faster ligation than cleavage. This enzyme ratio is constant for all further dynamic polymerizations, unless when studying the influence of the enzyme concentrations.

**Fig. 2:**
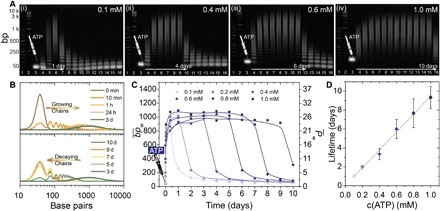
ATP-fueled transient DySS polymerization of dynamic covalent DNA chains with a tunable lifetime. (**A**) Time-dependent GE shows transient lifetimes programmed by ATP fuel concentration (0.1 to 1.0 mM ATP, left to right). Lane assignment: 1, 50-bp ladder; 2, 1-kbp ladder; 3, 0 min; 4, 10 min; 5, 1 hour; 6, 9 hours; 7, 24 hours; 8 to 16, 2 to 10 days (daily interval). (**B**) Gray scale profiles extracted from GE at 0.4 mM ATP (panel ii) quantify the transient, reversible shifts of molecular weight (top, growth; bottom, decline), which is used to calculate the mass-weighted average chain length (${\bar{\mathrm{bp}}}_{w}$) for each kinetic aliquot. (**C**) The development of ${\bar{\mathrm{bp}}}_{w}$ over time reveals increasing lifetimes of the transient DNA polymerization with a constant steady-state chain length of around 1000 bp under the given enzymatic conditions when increasing the ATP concentration from 0.1 to 1.0 mM. Lines are guides to the eye. (**D**) The lifetime scales linearly with the amount of supplied ATP. Error bars result from averaging triplicate measurements. Conditions: 0.05 mM M_1_, 41.25 WU of T4 DNA ligase, 900 U of BamHI, and varying amounts of ATP at 25°C in the enzyme reaction buffer.

Considering the importance of the chemical fuel in a dissipative system, we first discuss its influence on the ensemble system behavior of the transient DySS polymerization of dynamic covalent DNA chain growth (25°C). Experimentally, we analyze the time-dependent behavior from kinetic aliquots via agarose gel electrophoresis (GE; Fig. 2A). GE allows the resolution of the chain length distribution of the dynamically polymerizing M_1_-based DNA chains accurately, in particular with regard to smaller oligomers.

Close inspection of a system fueled, e.g., with 0.4 mM ATP reveals the monomer band (M_1_, 38 bp) at the bottom of lane 3 (*t* = 0; Fig. 2A, ii). Injection of ATP initiates chain growth rapidly, and the system enters the DySS (lanes 5 to 9), where continuous exchange (ligation/cutting) occurs. After 3 days, the chain length declines. A short oligomer fraction persists for extended time, as the degradation by BamHI eventually slows down due to depletion of the substrate according to Michaelis-Menten reaction kinetics. Analysis of the gray scale profiles of each lane allows quantification of the distributions and displays a shift to higher molecular weights at initial stages and back to M_1_ when the system runs out of fuel (Fig. 2B). Those distributions equal mass-weighted chain length distributions, which can be calibrated using DNA ladders to derive mass-weighted average chain lengths, ${\bar{\mathrm{bp}}}_{w}$ (Supplementary Note B, fig. S6).

Figure 2C illustrates the corresponding transient polymerization profiles using the calculated ${\bar{\mathrm{bp}}}_{w}$ for increasing ATP concentrations. Evidently, the lifetimes of the continuously ATP-dissipating DySS polymers extend from less than 1 day to ca. 10 days with increasing fuel levels. Both enzymes remain fully operational even for such extended durations (fig. S4). The ligation depends on the ATP concentration, as ATP is a fueling cosubstrate (*39*). The lifetimes are defined to the point where ${\bar{\mathrm{bp}}}_{w}$ declines from the DySS plateau value. They show a linear correlation with the ATP concentration, underscoring an excellent control over the temporal programmability of the transient DySS of the DNA chains (Fig. 2, C and D). Despite the different lifetimes, all systems evolve into the same plateau in the DySSs with ${\bar{\mathrm{bp}}}_{w}$ values of ca. 1000 bp, which equals an average degree of polymerization ${\bar{P}}_{w}$ of ca. 26, programmed by the balance between ligation and cleavage. Given the high persistence length of dsDNA (ca. 50 nm at 0.1 N NaCl), this corresponds to the formation of long semiflexible fibrils, with a diameter of 2.0 nm and a mass-average length, ${\bar{l}}_{w}$, of ca. 340 nm being similar to a range of mostly noncooperatively assembling supramolecular fibrils.

### Biocatalytic and thermal programming of structural and dynamic steady-state properties of the DNA polymers

**Fig. 3:**
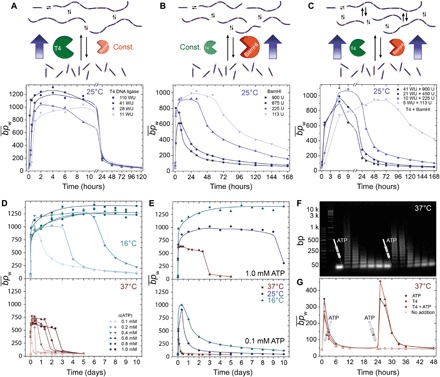
Programming the transient ATP-fueled DySS polymerization of DNA chains by changing the dynamics of ligation and cleavage under biocatalytic and thermal control. The starting configuration of the systems comprises 0.05 mM M_1_ (38 bp), 41.25 WU of T4 DNA ligase, 900 U of BamHI, and 0.1 mM ATP in the enzyme reaction buffer at 16°, 25°, and 37°C. Each of these parameters is systematically varied to tune the dynamics of the transiently evolving chains: Increase of (**A**) T4 DNA ligase, (**B**) BamHI, or (**C**) of both enzymes symmetrically shifts the kinetic balance of the competing reactions either to the ligation or restriction side, leading to different DySSs and lifetimes (0.1 mM ATP, 25°C). (**D**) Dynamics and ATP-dependent lifetimes can be further controlled by temperature: top, 16°C, dynamics slow down; bottom, 37°C, dynamics speed up. (**E**) Comparison of the temperature-dependent temporal development of the average chain length ${\bar{\mathrm{bp}}}_{w}$ for selected ATP concentrations: top, 1.0 mM; bottom, 0.1 mM. (**F**) Time-dependent GE showing reactivation of transient chain growth by addition of ATP (both cycles fueled with 0.1 mM ATP, 37°C). (**G**) The corresponding plots of ${\bar{\mathrm{bp}}}_{w}$ over time demonstrate identical dynamic system behavior for the second cycle. Control experiments elucidate ATP as the driving force for successful reinitiation. Lines in all graphs are drawn as a guide to the eye.

We hypothesized that the variation of the enzyme ratio could manipulate the DySS bond and the ensemble system behavior under biocatalytic control. To this end, we changed the T4 DNA ligase concentration ([T4]) while keeping the restriction enzyme concentration constant ([BamHI] = 900 U; Fig. 3A). The increase of [T4] from 11 to 110 WU has two main effects on the DySS polymers. First, it results in a faster buildup of the DySS (initial growth phase), and second, it leads to longer DNA chains with an increase of ${\bar{\mathrm{bp}}}_{w}$ from ca. 900 to 1200 bp. Note that the increase of T4 DNA ligase also affects the lifetime of the DySS, but this is not resolved in Fig. 3A as the focus lies on the inspection of the different DySS average chain lengths. Both effects can be explained by a shift of the kinetic balance toward the ligation side by its selective acceleration. Likewise, cleavage can be favored when increasing [BamHI] (113 to 900 U, Fig. 3B), while [T4] stays unchanged (41.25 WU). More frequent cleavage events shorten ${\bar{\mathrm{bp}}}_{w}$ and the lifetime of the DySS drastically. The transient DySS polymers degrade in the range of days faster for high concentrations of BamHI.

The intermolecular bond exchange dynamics in the DySS polymerization system can be accelerated by a symmetric increase (here up to 8×) of both enzymes at a fixed ratio [T4]/[BamHI] = 5 WU/113 U (Fig. 3C). This leads to narrower time profiles of the DySS polymerization with both faster chain growth and degradation and consequently to shorter lifetimes. Higher enzyme activities on both sides of the antagonistic reaction network mean faster conversion of ATP and higher exchange frequencies of the dynamic covalent bond. The possibility of adjusting the exchange frequencies within the DySS is instrumental regarding self-renewal/self-healing and adaptivity, and a unique advantage of this chemically fueled system with synchronized energetic and structural events.

The DySS polymerization can also be tuned by changing the temperature, which is particularly important to understand at near physiological conditions (37°C), as we operate a highly biocompatible system. Whereas BamHI shows higher activity at 37°C, the optimum temperature for the T4 DNA ligase is a trade-off between its activity and the hybridization probability of two 4-nt overhangs. Lower temperatures stabilize the complementary overhangs and thus facilitate ligation. This effect can be observed in the ATP-dependent DySS polymerization systems at 16°C (top) and 37°C (bottom; Fig. 3D). At 16°C, the chains evolve into a DySS with a ${\bar{\mathrm{bp}}}_{w}$ ≈ 1200 bp (${\bar{l}}_{w}$ ≈ 410 nm), hence almost twice as high compared to 37°C (${\bar{\mathrm{bp}}}_{w}$ ≈ 600 bp; ${\bar{l}}_{w}$ ≈ 200 nm). The lower temperature (16°C) favors the ligation, whereas the higher temperature (37°C) shifts the reaction balance to the restriction side. The second important point is the difference in the DySS lifetimes at a given ATP concentration (Fig. 3E). Because of reduced enzymatic reaction rates at low temperatures and thus slower ATP conversion, the lifetimes of the DySS at 16°C exceed those at 37°C (e.g., by more than several days at 1.0 mM ATP), as less energy is dissipated per time. At 16°C and [ATP] ≥ 0.6 mM, the DySS lifetime even exceeds the chosen experimental timeframe of 10 days. Since both enzymes are stable over time, this effect is clearly rooted in the slower conversion of ATP. The dispersity, Đ, of the DySS polymerization system follows step growth reaction kinetics (Đ = 1 + *p*; *p* = steady state conversion; dynamic bond ratio) and varies between 1 and 2 according to the DySS conversion in the system. It can be programmed by the effective enzymatic balance and temperature (fig. S7).

Critically, refueling experiments with a second addition of ATP after completion of one polymerization cycle underscore that aging of the enzymes plays no notable role within the investigated timeframe (Fig. 3, C and D, and figs. S4 and S8). The second cycle looks almost identical to the first one with respect to lifetime and average chain length (see also fluorescence experiments in fig. S11 with four consecutive activation cycles). Control experiments without ATP fail to initiate the second cycle and thereby confirm ATP clearly as the chemical driver of the DySS polymerization system. Overall, the ability to program DySS lifetimes up to weeks with high ATP concentrations following a linear dependence, to operate the system at different temperatures, and to reactivate several cycles confirms a very robust and long-living system with little problems concerning product inhibition (waste; AMP + PP_i_) or enzyme stability.

### Molecular dynamics and adaptation within the DySSs

**Fig. 4:**
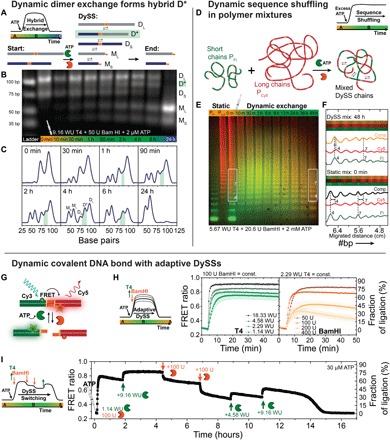
Adaptive DySSs and molecular exchange in the dynamic covalent DNA bond system. (**A** to **C**) Intermolecular exchange between two different dimer duplexes D_S_ (72 bp) and D_L_ (100 bp) upon enzymatic dynamization of the dynamic covalent restriction site. (A) DNA species formed during the transient ATP-fueled dynamization. (B) GE of ATP-fueled dimer exchange kinetics (37°C) shows the transient occurrence of a hybrid species D* (84 bp) and provides evidence for molecular reshuffling of the fragments. (C) Gray scale profiles highlight D* in green. Conditions: 0.5 μM D_L_, 0.5 μM D_S_, 37°C. (**D** to **F**) Dynamic sequence shuffling between fluorescently labeled DNA chains proves intermolecular subunit exchange also on the polymer level. (D) Two homopolymers, short fluorescein-tagged P_Fl_ (green) and long Cy5-tagged P_Cy5_ (red), were mixed together and turned into a random copolymer upon DySS activation. (E) The shuffling process and evolution into a DySS polymer is followed by selective multicolor GE. The multicolor GE is a composite image of the fluorescein (green) and the Cy5 (red) channel. The fluorescent oligomers show different migration distances and can be distinguished from each other (compare first two lanes of pure homopolymers P_Fl_ and P_Cy5_). A randomized DySS sequence appears in orange color and by homogenization of the band migration. (F) Gray scale analysis of the individual fluorophore channels and the composite reveals the different compositions of the static (0 hour) and dynamic (48 hours) polymer “mix” (framed sections in GE). Convergence of the initially separated bands into one DySS band, for instance, the heptamer fraction (no. 7), demonstrates successful sequence shuffling and subunit exchange. Conditions: 5.0 μM M_Fl_ in P_Fl_, 2.5 μM M_Cy5_ in P_Cy5_, 37°C. (**G** to **I**) Adaptive DySSs monitored by FRET duplex activation (fig. S9 and S10 for details). (G) The dynamic covalent bond was equipped with the Cy3/Cy5 FRET pair to report the DySS ligation level via the FRET ratio I_max,Cy5(acceptor)_/I_max,Cy3(donor)_. The FRET ratio can be translated into a fraction of ligation, which is effectively an ensemble average steady-state bond strength. (H) Formation of different DySSs in dependence of the enzyme ratio [T4]/[BamHI] at 25°C: variation of the T4 DNA ligase (left, [BamHI] = 100, U = constant) and BamHI (right, [T4] = 2.29, WU = const). (I) In situ adaptation of the DySS in a transient ATP-fueled FRET duplex activation by sequential addition of individual enzymes. Conditions: 1 μM F_Cy3_, 1 μM F_Cy5_, 25°C, λ_exc_ = 505 nm.

Last, we investigate more closely the detailed dynamics of the system to evidence and understand the intermolecular exchange and the adaptation within true DySSs. We start with a simplified system of two DNA duplex dimers of different lengths, D_L_ (100 bp) and D_S_ (72 bp), with an internal restriction site to demonstrate the molecular exchange of DNA fragments (Fig. 4A). Upon ATP-fueled enzymatic dynamization, the duplexes are continuously cleaved and recombine randomly, and thereby generate a new transient hybrid species D* of intermediate length (84 bp; Fig. 4, A and B). Gray scale analysis highlights the transient occurrence of the hybrid species D* in the DySS between 1 and 6 hours (D* in green) before everything is eventually cleaved into the monomeric fragments M_L_ and M_S_ (Fig. 4C). This confirms unambiguously intermolecular exchange between the DNA dimers and provides avenues to program transient functionality.

On a polymer level, intermolecular exchange and bond shuffling occurs constantly between dynamized DNA polymers. To visualize and understand this time-dependent process, we dynamized a mixture of two fluorescently labeled DNA polymers with an excess of ATP. Using multicolor GE imaging, Fig. 4 (D and F) illustrates how short fluorescein-labeled (P_Fl_) and long Cy5-labeled DNA polymers (P_Cy5_) undergo sequence randomization into a statistically mixed composition upon evolution of the DySS. At the beginning, individual fluorescent P_Fl_ and P_Cy5_ oligomers are distinguishable by different migration behaviors and colors in the composite GE image due to the influence of the attached fluorophores. However, upon bond shuffling in the DySS, the two initially separated red (P_Cy5_) and green (P_Fl_) static chain length distributions merge into a single mixed one of orange color, which adopts the DySS properties given by the specific enzymatic conditions. The disappearance of the oligomeric migration shift between the individual P_Fl_ and P_Cy5_ bands and the convergence into one band can be convincingly visualized via gray scale analysis of the individual fluorophore channels and the composite image at *t* = 0 hour (static mixture) and at *t* = 48 hours (DySS; Fig. 4F).

All experiments presented so far indicate that the [T4]/[BamHI] ratio dynamically controls the degree of steady-state ligation and the molecular exchange frequencies within the DySS. This should make the DySS systems highly adaptive to changes in the enzymatic environment. To allow an in situ readout of the adaptive behavior, we used a DNA duplex F (42 bp) equipped with the Cy3/Cy5 fluorescence resonance energy transfer (FRET) pair close to the internal restriction site. The FRET duplex F reports its DySS and the average steady-state bond strength of the ensemble by FRET-induced emission of the Cy5 acceptor dye, while the cleaved fragments F_Cy3_ and F_Cy5_ lack FRET (Fig. 4G). Spectral changes upon dynamization of the F_Cy3_ and F_Cy5_ fragments were evaluated by the FRET ratio (Cy5/Cy3 = I_674 nm_/I_571 nm_), which can be converted into a relative percentage of ligation, with greater precision and higher temporal resolution than in GE (details in fig. S10). Figure 4H demonstrates the evolution into programmable DySSs by variation of the enzyme ratio [T4]/[BamHI] at 25°C starting with the fully cleaved fragments. Increasing [T4] (1.14 to 18.33 WU) at a constant [BamHI] = 100 U (left) allows faster access to the DySS, and the extent of DySS ligation increases from ca. 63 to 83%. The stable plateau of the FRET ratio in the DySS confirms the development of true steady states with constant rates of ligation and cleavage. The degree of DySS ligation decreases drastically for higher [BamHI], as displayed in the right panel with a decrease down to ca. 34% at [BamHI] = 400 U, while [T4] = 2.29 WU is constant. Because of the rapid ATP conversion at this very high cleavage activity, the transient nature of the fueled system is visible with a final decay into the fully cleaved state.

Critically, the DySS ligation level adapts promptly to manipulations of the enzyme ratio as visualized by in situ monitoring of the DySS and stepwise addition of the individual enzymes, T4 DNA ligase or BamHI (Fig. 4I). Starting from 1.14 WU of T4 DNA ligase and 100 U of BamHI, the FRET duplex system is activated by 30 μM ATP and evolves into its first DySS with a dynamic ligation plateau of ca. 64%. Another addition of T4 DNA ligase (+9.16 WU) shifts the DySS balance more strongly toward the ligation side and increases the dynamic ligation ratio up to ca. 77%, while subsequent injections of BamHI (100 U) promote the cleavage and reduce the DySS plateau stepwise to ca. 31%. After each disturbance of the enzymatic balance, the system needs time for adaptation to form a new stable DySS. However, further manipulations of the DySS can be carried out until the system runs out of fuel (here, ca. 15 hours). Additional ATP-dependent lifetimes and refueling experiments monitored by FRET are in fig. S11. Overall, this repeated adaptation to different DySSs with full reversibility of the dynamically cleaved bond underscores the robustness and integrity of the system.

## DISCUSSION

In this work, we bridge the gap between stable, robust covalent structure formation and the programmable dynamics of kinetically controlled molecular exchange in nonequilibrium systems by introduction of a fuel-driven dynamic covalent bond system. In contrast to classic, sensitized, equilibrium-type dynamic covalent bonds, this dissipative system needs energy for making—and not for breaking—the covalent bond. This provides unprecedented controllability and inherent access to more complex, highly adaptive, and autonomous steady-state behavior. The key properties of such a chemically fueled dynamic covalent bond are isothermally controlled DySSs with programmable and adaptive fractions of the bound state (bond ratio), tunable exchange frequencies, and transient lifetimes of the ensemble on a systems level. Critically, the chemical fuel is only an energy-providing cofactor and only serves to power the bond formation between two functional partners and does not represent one of those. This provides the flexibility in molecular design, which is needed to access covalent connectivity patterns on larger length scales, of different topologies, and of emergent functionalities.

We investigated the structural implications of this by implementation of an ATP-fueled, enzymatically activated and dynamized DNA phosphodiester bond, which was used for the transient polymerization of short dsDNA monomers into DySS polymers. The integrated dissipative, dynamic covalent bond continuously consumes chemical energy by conversion of ATP, and the dynamics can be controlled by the kinetics of the enzymatic reaction network of ligation and cleavage. The availability of ATP mainly controls the lifetime of the dynamic polymers, while the absolute enzyme concentrations and the kinetic balance of ligation and cleavage regulate the average chain length and the exchange dynamics of the DySS. The system is completely reversible and can be reactivated by addition of fresh ATP, with waste products having little effect on reactivation.

Strikingly, this system features simultaneous programmability on a temporal, structural, and steady-state dynamics level in nonequilibrium molecular systems. A decisive advantage of the chemically fueled dynamic covalent bond is the fact that energetic events are merged with structural transitions and concurrently modulate the intermolecular dynamics of the ensemble, which is not possible for chemically fueled supramolecular system approaches. In addition, for the ATP-driven dynamic covalent DNA bond, the facile programmability of DNA systems and the availability of a large range of restriction enzymes will allow quick progress toward rational design of the behavior in nonequilibrium systems, including different life cycles and multicomponent systems, and allow spatiotemporal organizations of functions in general. The next steps on a materials level will be to translate this emergent behavior into programmable nonequilibrium structure/property relationships. The integration of this ATP-fueled dynamic covalent bond into DNA hybrid soft matter systems is highly appealing, e.g., for active DNA hydrogels with programmable and adaptive stress relaxation behavior to study fundamental cell behavior or for fueled self-healing via preorchestrated reshuffling of dynamic cross-links. We believe that chemically fueled dynamic covalent bond systems are an avenue for robust and deterministic dissipative nonequilibrium materials systems, and we are excited about finding further suitable coupling reactions that allow this behavior in other material classes.

## MATERIALS AND METHODS

### Hybridization of the DNA building blocks

The DNA monomer **M**_**1**_ was obtained by mixing the complementary DNA strands **M**_**a**_ and **M**_**b**_ (each from 1 mM stock in the annealing buffer) in a stoichiometric ratio. The mixture (0.5 mM) was annealed in a thermocycler by heating to 95°C for 2 min and then cooling down to 20°C with a controlled temperature rate of 0.01°C/s.

The fluorescently labeled DNA duplex strands **F**, **D**_**S**_, **D**_**L**_, **M**_**Fl**_, and **M**_**Cy5**_ were hybridized stoichiometrically in the 1× reaction buffer E from their single-stranded constituents a and b by incubation at 37°C for 1 hour to give a final storage solution of 25 μM dsDNA. Hybridized DNA stock solutions were stored at −20°C. All oligonucleotide sequences are listed in table S1.

### Transient dynamic DNA polymerization system

Enzymatic reactions of the dynamic chain growth were typically assembled in a total reaction volume of 90 μl as follows: Sterile water, DNA M_1_, 10× buffer E, bovine serum albumin (BSA), T4 DNA ligase, and the BamHI restriction enzyme were added sequentially in a polymerase chain reaction tube. The solution was mixed gently by pipetting up and down, and centrifuged shortly before addition of the ATP to initiate the reaction system. The enzymatic reaction was incubated in a thermoshaker at 250 rpm. Incubation temperatures (16°, 25°, and 37°C) and the concentrations of the enzymes and the ATP (0.1 to 1.0 mM) varied depending on the experiment and are stated at the corresponding figures. The concentrations of all other components [0.05 mM DNA, 1× buffer E, and BSA (0.1 g/liter)] were kept constant in the reaction mixture throughout all kinetic assays.

Time-dependent aliquots (6 μl) were withdrawn from the reaction tube and immediately quenched in the quenching buffer containing EDTA and subsequent freezing in liquid nitrogen. Time intervals were adapted to the kinetics of the experiments to follow the reaction progress appropriately.

Kinetic aliquots were analyzed by electrophoretic mobility shift assays. GE was carried out in 2 weight % agarose gels in tris-acetate-EDTA buffer applying 90 V = constant, 300 mA, 90 min using in-cast staining with Roti-GelStain.

## Supplementary Material

http://advances.sciencemag.org/cgi/content/full/5/7/eaaw0590/DC1

Download PDF

## SUPPLEMENTARY MATERIALS

Supplementary material for this article is available at http://advances.sciencemag.org/cgi/content/full/5/7/eaaw0590/DC1 Supplemental Materials and Methods Experimental Protocols Supplementary Note A. Development of the conditions for the dynamic reaction network by characterization of the individual enzyme reactions Supplementary Note B. Routine of GE analysis: From the agarose gel to an average chain length Supplementary Note C. ATP-fueled transient, dynamic steady-state DNA polymerization system Supplementary Note D. DySS and molecular exchange in ATP-fueled dissociative dynamic covalent DNA systems Table S1. Oligonucleotide sequences. Fig. S1. Hybridization of the self-complementary ends of the DNA monomer strands M_1_ in dependence of temperature and ligation reaction catalyzed by T4 DNA ligase. Fig. S2. Ligation kinetics of the DNA chain growth as a function of T4 DNA ligase concentration. Fig. S3. Ligation kinetics of the DNA chain growth as a function of ATP concentration. Fig. S4. Time-dependent T4 DNA ligase catalyzed ligation reaction. Fig. S5. Restriction kinetics of the DNA chain cleavage as a function of BamHI concentration. Fig. S6. Routine for analysis of GE data: From the agarose GE to an average DNA chain length ${\bar{\mathrm{bp}}}_{w}$. Fig. S7. Control of dispersity in the DySS DNA polymerization system. Fig. S8. Refueling experiments of the transient DySS DNA polymerization system. Fig. S9. Average chain length in the transient DySS DNA polymerization system in dependence of the concentration of the DNA monomer M_1_. Fig. S10. Characterization of the FRET duplex F and its cleaved and religated DNA fragments as used for in situ modulation of the DySS. Fig. S11. ATP-dependent temporal control of the dynamic DNA bond with transient DySS FRET duplex formation. References (*40*, *41*)
