# Supplementary material for: Programmable dynamic steady states in ATP-driven nonequilibrium DNA systems
Source: Sci Adv. 2019 Jul 19;5(7):eaaw0590. doi: 10.1126/sciadv.aaw0590 (PMC6641946; doi:10.1126/sciadv.aaw0590)
Supplement: http://advances.sciencemag.org/cgi/content/full/5/7/eaaw0590/DC1 [file supp_5_7_eaaw0590__index.html]

Science Advances | Science AdvancesAAASSearchScience AdvancesMenu

## Supplementary Materials

**This PDF file includes:**

- Supplemental Materials and Methods
- Experimental Protocols
- Supplementary Note A. Development of the conditions for the dynamic reaction network by characterization of the individual enzyme reactions
- Supplementary Note B. Routine of GE analysis: From the agarose gel to an average chain length
- Supplementary Note C. ATP-fueled transient, dynamic steady-state DNA polymerization system
- Supplementary Note D. DySS and molecular exchange in ATP-fueled dissociative dynamic covalent DNA systems
- Table S1. Oligonucleotide sequences.
- Fig. S1. Hybridization of the self-complementary ends of the DNA monomer strands M1 in dependence of temperature and ligation reaction catalyzed by T4 DNA ligase.
- Fig. S2. Ligation kinetics of the DNA chain growth as a function of T4 DNA ligase concentration.
- Fig. S3. Ligation kinetics of the DNA chain growth as a function of ATP concentration.
- Fig. S4. Time-dependent T4 DNA ligase catalyzed ligation reaction.
- Fig. S5. Restriction kinetics of the DNA chain cleavage as a function of BamHI concentration.
- Fig. S6. Routine for analysis of GE data: From the agarose GE to an average DNA chain length bp¯w.
- Fig. S7. Control of dispersity in the DySS DNA polymerization system.
- Fig. S8. Refueling experiments of the transient DySS DNA polymerization system.
- Fig. S9. Average chain length in the transient DySS DNA polymerization system in dependence of the concentration of the DNA monomer M1.
- Fig. S10. Characterization of the FRET duplex F and its cleaved and religated DNA fragments as used for in situ modulation of the DySS.
- Fig. S11. ATP-dependent temporal control of the dynamic DNA bond with transient DySS FRET duplex formation.
- References (*40*, *41*)

Download PDF

**Files in this Data Supplement:**

- Adobe PDF - aaw0590\_SM.pdf
