# Supplementary material for: Programmable dynamic steady states in ATP-driven nonequilibrium DNA systems
Source: Sci Adv. 2019 Jul 19;5(7):eaaw0590. doi: 10.1126/sciadv.aaw0590 (PMC6641946; doi:10.1126/sciadv.aaw0590)
Supplement: Download PDF [file aaw0590_SM.pdf]

Science Advances
AAAS

advances.sciencemag.org/cgi/content/full/5/7/eaaw0590/DC1

Supplementary Materials for

Programmable dynamic steady states in ATP-driven nonequilibrium DNA systems

Laura Heinen and Andreas Walther*

*Corresponding author. Email: andreas.walther@makro.uni-freiburg.de

Published 19 July 2019, *Sci. Adv.* **5**, eaaw0590 (2019)
DOI: 10.1126/sciadv.aaw0590

This PDF file includes:

Supplemental Materials and Methods
Experimental Protocols
Supplementary Note A. Development of the conditions for the dynamic reaction network by characterization of the individual enzyme reactions
Supplementary Note B. Routine of GE analysis: From the agarose gel to an average chain length
Supplementary Note C. ATP-fueled transient, dynamic steady-state DNA polymerization system
Supplementary Note D. DySS and molecular exchange in ATP-fueled dissociative dynamic covalent DNA systems
Table S1. Oligonucleotide sequences.
Fig. S1. Hybridization of the self-complementary ends of the DNA monomer strands M_1 in dependence of temperature and ligation reaction catalyzed by T4 DNA ligase.
Fig. S2. Ligation kinetics of the DNA chain growth as a function of T4 DNA ligase concentration.
Fig. S3. Ligation kinetics of the DNA chain growth as a function of ATP concentration.
Fig. S4. Time-dependent T4 DNA ligase catalyzed ligation reaction.
Fig. S5. Restriction kinetics of the DNA chain cleavage as a function of BamHI concentration.
Fig. S6. Routine for analysis of GE data: From the agarose GE to an average DNA chain length $\overline{bp}_w$.
Fig. S7. Control of dispersity in the DySS DNA polymerization system.
Fig. S8. Refueling experiments of the transient DySS DNA polymerization system.
Fig. S9. Average chain length in the transient DySS DNA polymerization system in dependence of the concentration of the DNA monomer M_1.
Fig. S10. Characterization of the FRET duplex F and its cleaved and religated DNA fragments as used for in situ modulation of the DySS.
Fig. S11. ATP-dependent temporal control of the dynamic DNA bond with transient DySS FRET duplex formation.
References (*40*, *41*)

Selected Recurring Abbreviations

**ssDNA**	single-stranded DNA
**dsDNA**	double-stranded DNA
**GE**	gel electrophoresis
**nt**	nucleotide
**bp**	base pair
**kbp**	kilo base pair
**DySS**	dynamic steady state
**FRET**	Förster resonance energy transfer

Supplemental Materials and Methods

Instrumentation:

TSC Thermoshaker (Analytik Jena), Thermocycler Tpersonal (Analytik Jena), U:Genius^3 Gel electrophoresis documentation system (Syngene), UVsolo *touch* Gel electrophoresis documentation system (Analytik Jena), gel electrophoresis chambers (biostep), power source 250 V (VWR), Enduro power supply 300 V (Labnet International, Inc.), Large blue LED transilluminator (470 nm, IORodeo), Home-built RGB-LED-Transilluminator (Red: 620-630 nm, Green: 515-530 nm, Blue: 465-475 nm), Band pass filters: 545BP40-50x50 (Laser Components), 690BP40-50x50 (Laser Components), 605BP50-50x50 (Analytik Jena). Image J 1.51k, ScanDrop® UV-VIS spectrometer (Analytik Jena), QE *Pro* spectrometer with fluorescence measurement set-up (Ocean Optics), fiber coupled LED light source 505 nm (LLS series, Ocean Optics), CUV-QPOD Temperature Controlled Cuvette Holder qpod 2e™ (Ocean Optics).

Reagents:

The following chemicals, reagents and enzymes were used. Ethylenediaminetetraacetic acid disodium salt dihydrate (EDTA, biology grade, CALBIOCHEM), sodium chloride (NaCl, 99%, ABCR), tris (hydroxymethyl)aminomethane hydrochloride pH 8.8 and pH 8.0 (Tris-HCl, Trizma buffer substance pH 8.8 and pH 8.0, Sigma-Aldrich), Trizma base (Sigma-Aldrich), Acetic acid glacial (ACS, Reag. Ph. Eur. analytical reagents, VWR Chemicals), Gene Ruler 1kb DNA ladder (ready-to-use, Thermo Scientific, fragments [bp]: 250, 500, 750, **1000**, 1500, 2000, 2500, **3000**, 3500, 4000, 5000, **6000**, 8000, 10000), Gene Ruler 50bp DNA ladder (ready-to-use, Thermo Scientific, fragments [bp]: 50, 100, 150, 200, **250**, 300, 400, **500**, 600, 700, 800, 900, 1000), TrackIT™ Ultra Low Range DNA ladder (ready-to-use, Invitrogen, fragments [bp]: 10, 20, 35, 50, 75, **100**, 150, 200, 300), Roti®-GelStain (1,1'3,3',5,5'6,6'-Octamethyl-2,2'-spiro(2,3-dihydro-1H-Benzimidazol), Carl Roth), SYBR™ Gold Nucleic Acid Gel Stain (10000x Concentrate in DMSO, Invitrogen), T4 DNA Ligase in Storage Buffer (20 WU/µl (HC) and 3 WU/µl , source: recombinant *E. coli* strain, Promega), BamHI in Storage Buffer (80 U/µl (HC) and 10 U/µl, source: *Bacillus amyloliquefaciens* H, Promega), 10x Buffer E (Promega), ATP solution (10 mM in 1 mM Tris-HCl pH 7.5, Invitrogen), ATP Solution Tris-buffered (100 mM, pH 7.3-7.5,

Thermo Scientific), Agarose low EEO (Agarose Standard, PanReac AppliChem), Acetylated Bovine Serum Albumine (BSA, 10 g/L, Promega), Glycerol (Ultrapure, Invitrogen).

Buffer compositions:

T4 DNA Ligase Storage Buffer (Promega): 10 mM Tris-HCl (pH 7.4 at 25 °C), 50 mM KCl, 1 mM Dithiothreitol (DTT), 0.1 mM EDTA, 50% glycerol.
BamHI Storage Buffer (Promega): 10 mM Tris-HCl (pH 7.4), 300 mM KCl, 5 mM $MgCl_2$, 0.1 mM EDTA, 1 mM DTT, 0.5 mg/mL BSA, 50% glycerol.
Restriction Enzyme 10x Buffer E (Promega): 60 mM Tris-HCl (pH 7.5), 1 M NaCl, 60 mM $MgCl_2$, 10 mM DTT.
Annealing Buffer: 10 mM TRIS-HCl (pH 8.0), 50 mM NaCl.
TAE Buffer: 40 mM TRIS, 20 mM Acetic Acid, 1 mM EDTA.
Quenching Buffer: 200 mM EDTA, 10 mM TRIS-HCl (pH 8.0), 50 mM NaCl.

Oligonucleotides:

Oligonucleotides were purchased from Integrated DNA Technologies Inc. (USA, Belgium) in HPLC purity. DNA stock solutions were prepared by resuspending the lyophilized oligonucleotides in annealing buffer, unless for the fluorescently labeled strands which were directly dissolved in the 1x reaction buffer E. The FRET duplex strands F_a and F_b were synthesized by biomers.net GmbH (Germany). Sterile ultrapure water with conductivity less than 0.055 mS/cm was used throughout all experiments. Table S1 presents an overview of the used DNA strands. Note the phosphorylation at the 5'-end.

Table S1. Oligonucleotide sequences.

ID	oligonucleotide sequence	#nt
M_a	5'-/5Phos/GATC CTC TAT TCG CAT GAG AAT TCC ATT CAC CGT AAG G-3'	38
M_b	5'-/5Phos/GATC CCT TAC GGT GAA TGG AAT TCT CAT GCG AAT AGA G-3'	38
D_{Sa}	5'-CTCTATTCGCATGAGAATTCCATTCACCGTAAGGGATCCTC/i6-TAMN/ ATTCGCATGAGAATTCCATTCACCGTAAGG-3'	72
D_{Sb}	5'-CCTTACGGTGAATGGAATTCTCATGCGAATAGAGGATCCCT/i6-TAMN/ ACGGTGAATGGAATTCTCATGCGAATAGAG-3'	72
D_{La}	5'- CGACTCTAGAGCGCCTCTATTCGCATGAGAATTCCATTCACCGTAAGGGATCCTC/iFlu orT/ ATTCGCATGAGAATTCCATTCACCGTAAGG CTGGCCGCTGTCGA-3'	100
D_{Lb}	5'-TCGACAGCGGCCAGCCTTACGGTGAATGGAATTCTCATGCGAATAGAGGATCCCT /iFluorT/ACGGTGAATG GAATTCTCATGCGAATAGAGGCGCTCTAGAGTCG-3'	100
F_a	5'-CATTCCATTCACCGTAAGGGATCCTC/dT-Cy3/ATTCGCATGAGAATT-3'	42
F_b	5'-AATTCTCATGCGAATAGAGGATCCCT/dT-Cy5/ACGGTGAATGGAATG-3'	42
M_a-Fl	5'-/5Phos/GATCCTCTATTCGCATGAGAA/iFluorT/TCCATTCACCGTAAGG-3'	38
M_b-Cy5	5'-/5Phos/GATCCCTTACGGTGAATGGAA/iCy5/TTCTCATGCGAATAGAG-3'	38

Experimental Protocols

Ligation kinetics of the DNA chain growth as a function of T4 DNA ligase concentration. The ligation assay was carried out as described in the manuscript for the transient dynamic system at 25 °C, but omitting the BamHI restriction enzyme. The amount of T4 DNA ligase varied from 5.5 WU to 110 WU with 1.0 mM ATP in the reaction mixture. See fig. S2.

Ligation kinetics of the DNA chain growth as a function of ATP concentration. The ATP-dependent ligation assay was carried out as described in the manuscript for the transient dynamic system at 25 °C, but omitting the BamHI restriction enzyme. T4 DNA ligase activity was set constant to 41.25 WU, while the ATP concentration varied from 0.001 mM to 1.0 mM in the reaction mixture. See fig. S3.

Long-term ligation kinetics of the DNA chain growth. The long-term ligation assay was carried out as described in the manuscript for the transient dynamic system at 25 °C, but omitting the BamHI restriction enzyme. T4 DNA ligase activity was set to 41.25 WU with 1.0 mM ATP in the reaction mixture. Kinetic aliquots were taken over a period of 9 days to check for maximum DNA chain length development and conversion. See fig. S4.

Activity assay of T4 DNA ligase over time to check for potential ageing effects. T4 DNA ligase (41.25 WU, 0.46 WU/µl) was incubated for a period of 10 days under the standard reaction conditions of the ligation reaction (V_{tot} = 90 µl, 1x buffer E, 0.1 g/L BSA) at 25 °C and 250 rpm. Ligation efficiency of this T4 DNA ligase stock was tested every 24 h by taking a small aliquot (8 µl), which was then mixed with fresh DNA M_1 (0.05 mM) and fresh ATP (1.0 mM). The mixture was reacted for 30 min (25 °C, 250 rpm) and subsequently quenched in quenching buffer by EDTA. The time-dependent ligation reactions were analyzed by GE as described above for the transient dynamic DNA chain growth reaction. See fig. S4.

Restriction kinetics of the DNA chain cleavage reaction as a function of BamHI concentration. First, the DNA substrate of the enzymatic digestion assay of BamHI was prepared by ligation of the monomer fragment, M_1, to maximum conversion into long DNA chains. The ligation reaction was typically assembled in a total volume of 50 µl by sequential addition of water, hybridized DNA (M_1, 0.1 mM), 10x buffer E (1x dilution), BSA (0.1 g/L) and T4 DNA ligase (50 WU, 1 WU/µl). The solution was mixed gently by pipetting up and down and centrifuged shortly before addition of the ATP (1 mM) to initiate the ligation reaction. The ligation reaction was incubated in a thermoshaker at 25 °C and 250 rpm for 24 h and subsequently the T4 DNA ligase was heat-deactivated at 70 °C for 10 min.
Several substrate batches of the ligated DNA chains were combined and carefully mixed to ensure the same starting material for every restriction assay. The kinetic assays of the cleavage reaction were assembled in a total volume of 90 µl by sequential addition of water, ligated DNA substrate (equivalent to 0.05 mM M_1), 10x buffer E (1x dilution) and BSA (0.1 g/L). The solution was mixed gently by pipetting up and down and centrifuged shortly before addition of the BamHI restriction enzyme to start the cleavage reaction. The concentration of the BamHI restriction enzyme varied from 450 U (5 U/µl) to 1800 U (20 U/µl). The enzymatic restriction reaction was incubated in a thermoshaker at 25 °C and 250 rpm and kinetic aliquots (6 µl) were taken at predetermined time intervals. These aliquots were treated and analyzed according to the

procedure described for the transient dynamic DNA chain growth system (cf. Methods and Materials in manuscript and Supplementary Note B). See fig. S5.

Refueling experiments of the transient dynamic DNA polymerization reaction system. The transient dynamic system was conducted as described in the manuscript using 41.25 WU T4 DNA ligase (0.46 WU/µl), 900 U BamHI restriction enzyme (10 U/µl) and 0.1 mM ATP as fuel. After completion of the first lifecycle with recovery of the original monomer sequence, the system was refueled by injection of either (i) ATP, (ii) T4 DNA ligase, (iii) ATP and T4 DNA ligase or (iv) no reagents added. The concentrations of the second injections were adjusted to match the conditions of the first cycle, corresponding to the reduced reaction volume due to withdrawn kinetic aliquots. Refueling of a second lifecycle is only successful upon addition of ATP. The refueling experiments were performed at 25 °C and 37 °C. See fig. S8 and Fig. 3F,G in the main manuscript.

Dynamic dimer exchange between different DNA duplexes monitored by GE. Two differently sized DNA duplexes $\mathbf{D_L}$ (fluorescein-labeled, 100 bp) and $\mathbf{D_S}$ (TAMRA-labeled, 72 bp), each having the BamHI restriction site positioned in the center of the sequence, were assembled in an equimolar ratio (1:1) in a PCR reaction tube by mixing water, 10x buffer E (1x in reaction), duplex $\mathbf{D_L}$ (0.5 µM) and $\mathbf{D_S}$ (0.5 µM), BSA (0.1 g/L), ATP (2 µM), T4 DNA ligase (9.16 WU) and varying amounts of BamHI restriction enzyme (50 U, 100 U and 200 U) to give a final reaction volume of 200 µl. The solution was mixed gently by pipetting up and down and centrifuged shortly before being incubated in a thermoshaker at 37 °C at 300 rpm. Time-dependent aliquots (20 µl) were withdrawn and immediately quenched in quenching buffer and subsequent freezing in liquid nitrogen. Kinetic aliquots were analyzed by GE in 4 wt% agarose gels in TAE buffer applying 75 V = const., 300 mA, 4 h and then poststained with SYBR gold for imaging. See Fig. 4A-C in the main manuscript.

Dynamic sequence shuffling of two different DNA homopolymers into one statistic copolymer and visualization by selective fluorophore GE imaging. First, two batches of DNA homopolymer chains with different degree of polymerization and different dye labels were prepared. Short, fluorescein-labeled DNA chains were obtained by ligation of $\mathbf{M_{Fl}}$ (10 µM) with 0.46 WU/µl T4 DNA ligase, 0.1 g/L BSA in 1x buffer E and <u>substoichiometric</u> ATP (3 µM) for 40 min at 25 °C to intentionally limit conversion to low molecular weights (V_{tot} = 150 µl). The T4 DNA ligase/DNA polymer solution was heat-deactivated (10 min, 70 °C) and spin filtered (3 kDa) with 1x buffer E to deactivate T4 DNA ligase and remove unreacted ATP.
Likewise, long, Cy5-labeled DNA chains were prepared by ligation of $\mathbf{M_{Cy5}}$ (10 µM) with 0.46 WU/µl T4 DNA ligase, 0.1 g/L BSA in 1x buffer E and <u>excess</u> ATP (1 mM) for 39 h at 25 °C to achieve maximum conversion (V_{tot} = 150 µl). The T4 DNA ligase/DNA polymer solution was heat-deactivated (10 min, 70 °C) and spin filtered (100 kDa) with 1x buffer E to deactivate T4 DNA ligase and remove unreacted ATP, as well as small DNA oligomers.
The dynamic sequence shuffling experiment was carried out in a total volume of 55 µl containing both DNA homopolymers (5 µM of short, fluorescein-labeled DNA chains, $\mathbf{P_{Fl}}$, and 2.5 µM of long, Cy5-labeled DNA chains, $\mathbf{P_{Cy5}}$; molar concentrations relative to the monomer content) with 2 mM ATP, 20.6 U BamHI and 5.67 WU T4 DNA ligase, 0.1 g/L BSA in 1x buffer E at 37 °C.

Time-dependent aliquots (6 µl) were withdrawn from the reaction tube and immediately quenched in quenching buffer containing EDTA and subsequent freezing in liquid nitrogen. Kinetic aliquots were analyzed by GE in 2 wt% agarose gels in TAE buffer, 90 V = const., 300 mA, 2.5 h.
The gel was analyzed by selective fluorophore imaging. The fluorescein-labeled DNA was imaged with excitation using a blue LED (ca. 465-475 nm) and a band-pass filter centered at 545 nm (BP 40 nm), while excitation with a red LED (ca. 620-630 nm) and a band-pass filter at 690 nm (40 nm) was used for the Cy5-labeled DNA. The DNA molecular weight ladders were visualized using the blue LED and a band-pass filter at 605 nm (50 nm) after post-staining with SYBR gold.
The gel images were stacked and processed in ImageJ, including correction of background, brightness, contrast and coloring, to obtain a multi-color composite image of both dyes, and further analyzed by gray scale profiling. See Fig. 4F in the main manuscript.

Enzymatic cleavage of the FRET duplex (F) with BamHI. In a typical cleavage reaction, the hybridized FRET duplex **F** (25 µM) was incubated with BSA (0.1 g/L) and the restriction enzyme BamHI (8 U/µl) in 1x buffer E at 37 °C, 300 rpm for 48 h. Full cleavage was verified by GE (60 min, 90 V = const., 300 mA, 4 wt% agarose stained with Roti-GelStain), and the sample was characterized by fluorescence spectroscopy (1 µM **F**, λ_{exc} = 505 nm, 1 s integration time, 25 °C). After heat-deactivation of the BamHI (10 min, 70 °C) the cleaved FRET duplex was used as starting material for the DySS FRET experiments. See fig. S10.

Enzymatic religation of the cleaved FRET duplex fragments with T4 DNA ligase. T4 DNA ligase (2 U/µl) and ATP (1 mM) were added to the heat-deactivated, cleaved FRET duplex solution (25 µM) and incubated at 25 °C, 300 rpm for 48 h. Subsequently, T4 DNA ligase was heat-deactivated (10 min, 70 °C). Full religation was verified by GE (60 min, 90 V = const., 300 mA, 4 wt% agarose stained with Roti-GelStain). The sample was characterized by fluorescence spectroscopy (1 µM cleaved **F**, λ_{exc} = 505 nm, 1 s integration time, 25 °C). See fig. S10.

Cleavage kinetics of the FRET duplex (F) with 100 U BamHI at 25 °C as monitored by FRET in fluorescence spectroscopy. The enzymatic cleavage reaction was assembled in a total volume of 200 µl by sequential addition of water, FRET duplex **F** (1 µM), 10x buffer E (1x dilution), BSA (0.2 g/L) and mixed gently by pipetting up and down. The overall glycerol content was typically kept at 5 vol%. The solution was transferred into a quartz glass cuvette (d = 3 mm) and equilibrated at 25 °C in the holder of the fluorescence spectrometer. The reaction was started by addition of BamHI (100 U, 0.5 U/µl). Cleavage of the FRET duplex was monitored by time-dependent emission spectra (λ_{exc} = 505 nm, 1 s integration time, 25 °C). See fig. S10.

Religation kinetics of the cleaved FRET duplex fragments with 4.58 WU T4 DNA ligase at 25 °C as monitored by FRET in fluorescence spectroscopy. The enzymatic ligation reaction was assembled in a total volume of 200 µl by sequential addition of water, the cleaved fragments of the FRET duplex **F** (1 µM), 10x buffer E (1x dilution), BSA (0.2 g/L), and mixed gently by pipetting up and down. The overall glycerol content was typically kept at 5 vol%. The solution was transferred into a quartz glass cuvette (d = 3 mm) and equilibrated at 25 °C in the holder of the fluorescence spectrometer. The reaction was started by addition of T4 DNA ligase (4.58 WU,

0.023 WU/µl) and ATP (4 µM). Religation of the FRET duplex was monitored by time-dependent emission spectra (λ_{exc} = 505 nm, 1 s integration time, 25 °C). See fig. S10.

Enzymatic control over the DySSs of the ATP-fueled dynamic covalent DNA bond of the FRET duplex as monitored by FRET in fluorescence spectroscopy. The DySS jump experiments presented in Fig. 4H of the main manuscript were assembled in a total volume of 200 µl by sequential addition of water, the cleaved fragments of the FRET duplex **F** (1 µM), 10x buffer E (1x dilution), BSA (0.2 g/L), and mixed gently by pipetting up and down. The overall glycerol content was typically kept at 5 vol%. The solution was transferred into a quartz glass cuvette (d = 3 mm) and equilibrated at 25 °C in the holder of the fluorescence spectrometer. The enzymes were added after 5 min in varying ratios: (Series A) BamHI = 100 U + varying T4 DNA ligase, (Series B) T4 DNA ligase = 2.29 WU + varying BamHI. After 5 min of equilibration, the reaction was started by addition of ATP (4 µM). Temporal evolution of the DySS of the FRET signal was monitored by time-dependent emission spectra (λ_{exc} = 505 nm, 1 s integration time, 25 °C). See Fig. 4H in the main manuscript.

In-situ adaptation of different DySSs of the dynamic covalent DNA bond in the FRET duplex by variation of the enzyme ratio as monitored by FRET in fluorescence spectroscopy. The DySS adaptation experiment of the dynamically ligated FRET duplex was assembled in a total volume of 200 µl by sequential addition of water, the cleaved fragments of the FRET duplex **F** (1 µM), 10x buffer E (1x dilution), BSA (0.2 g/L) and mixed gently by pipetting up and down. The overall glycerol content was typically kept at 5 vol%. The solution was transferred into a quartz glass cuvette (d = 3 mm) and equilibrated at 25 °C in the holder of the fluorescence spectrometer. Both enzymes, BamHI (100 U) and T4 DNA ligase (1.14WU) were added after 5 min of incubation. After another 5 min of equilibration, the reaction was started by addition of ATP (30 µM). The extent of dynamic ligation (visible as FRET pair formation) was controlled by addition of the respective enzymes – either T4 DNA ligase or BamHI – to switch between different DySSs: (1st addition) 9.16 WU T4 DNA ligase, (2nd add.) 100 U BamHI, (3rd add.) 100 U BamHI, (4th add.) 4.58 WU T4 DNA ligase, (5th add.) 9.16 WU T4 DNA ligase. Temporal evolution of the DySSs of the dynamic FRET bond was monitored by time-dependent emission spectra (λ_{exc} = 505 nm, 1 s integration time, 25 °C). See Fig. 4I in the main manuscript.

Lifetime control of the transient, dynamic FRET duplex ligation in dependence of ATP. The transient, dynamic ligation of the FRET duplex (= dynamic covalent bond formation) with an ATP-dependent lifetime was assembled in a total volume of 200 µl by sequential addition of water, the cleaved fragments of the FRET duplex **F** (1 µM), 10x buffer E (1x dilution), BSA (0.2 g/L), and mixed gently by pipetting up and down. The overall glycerol content was typically kept at 5 vol%. The solution was transferred into a quartz glass cuvette (d = 3 mm) and equilibrated at 25 °C in the holder of the fluorescence spectrometer. The enzymes, BamHI (100 U) and T4 DNA ligase (18.33 WU), were added after 5 min. After another 5 min of equilibration, the reaction was started by addition of varying amounts of ATP (0.5 µM – 20 µM). Temporal evolution of the DySS DNA bond was monitored by the transient FRET signal in time-dependent emission spectra (λ_{exc} = 505 nm, 1 s integration time, 25 °C). See fig. S11.

Refueling of dynamic FRET duplex ligation by multiple additions of ATP. Multiple repetitions of the transient, dynamic ligation of the FRET duplex (= dynamic covalent bond formation) were carried out in a total volume of 200 µl. The reaction solution was assembled by sequential addition of water, the cleaved fragments of the FRET duplex **F** (1 µM), 10x buffer E (1x dilution), BSA (0.2 g/L) and mixed gently by pipetting up and down. The overall glycerol content was typically kept at 5 vol%. The solution was transferred into a quartz glass cuvette (d = 3 mm) and equilibrated at 25 °C in the holder of the fluorescence spectrometer. The enzymes, BamHI (100 U) and T4 DNA ligase (4.58 WU), were added after 5 min. After another 5 min of equilibration, the reaction was started by addition of ATP (2 µM). After completion of one such transient dynamic ligation cycle, the reaction was refueled by addition of ATP (2 µM) several times. Temporal evolution of the dissociative DySS bond was monitored by the transient FRET signal in time-dependent emission spectra (λ_{exc} = 505 nm, 1 s integration time, 25 °C). See fig. S11.

Supplementary Note A. Development of the conditions for the dynamic reaction network by characterization of the individual enzyme reactions

Both the T4 DNA ligase and the BamHI restriction enzyme-catalyzed reactions were characterized independently of each other with the DNA monomer M_1 and its ligated polymer as substrates to establish suitable reaction conditions for the ATP-fueled dynamic polymerization system. We analyze different reaction parameters, involving the influence of enzyme, DNA and ATP concentration, reaction time and temperature, and enzyme stability, and discuss their effect on a step-growth-like polymerization in the following. Methodological details on GE analysis are provided in Supplementary Note B.

1.1. Hybridization Efficiency and Melting Behavior of the Self-Complementary Ends of Monomer Strand M_1

The transient DySS polymerization is conditional on the fact that the monomers may only polymerize in dependence of energy input via a chemically fueled reaction. Therefore, the self-complementary ends, which serve as telechelic end groups of the DNA monomer strands, M_1, are intentionally kept short to prevent uncontrolled self-hybridization and elongation into polymer chains (fig. S1A, B). The NUPACK(40) prediction of the melting profile of the 4 bp DNA hybridization (GATC) underpins that the self-complementary ends are largely unpaired under our reaction conditions (16 – 37 °C).

Figure S1C describes the ATP-dependent ligation reaction in general terms and fig. S1D shows greater mechanistic details of the enzyme-catalyzed phosphodiester bond formation. With regard to later stoichiometry and energy considerations, it is important to note that complete joining of the ssDNA overhangs of two DNA monomers consumes two molecules of ATP because of nick sealing in both strands of the duplex. Note that the fuel is not incorporated into the final structure, but only mediates bond formation as energy source. This provides important flexibility in the design of partners to be joined.

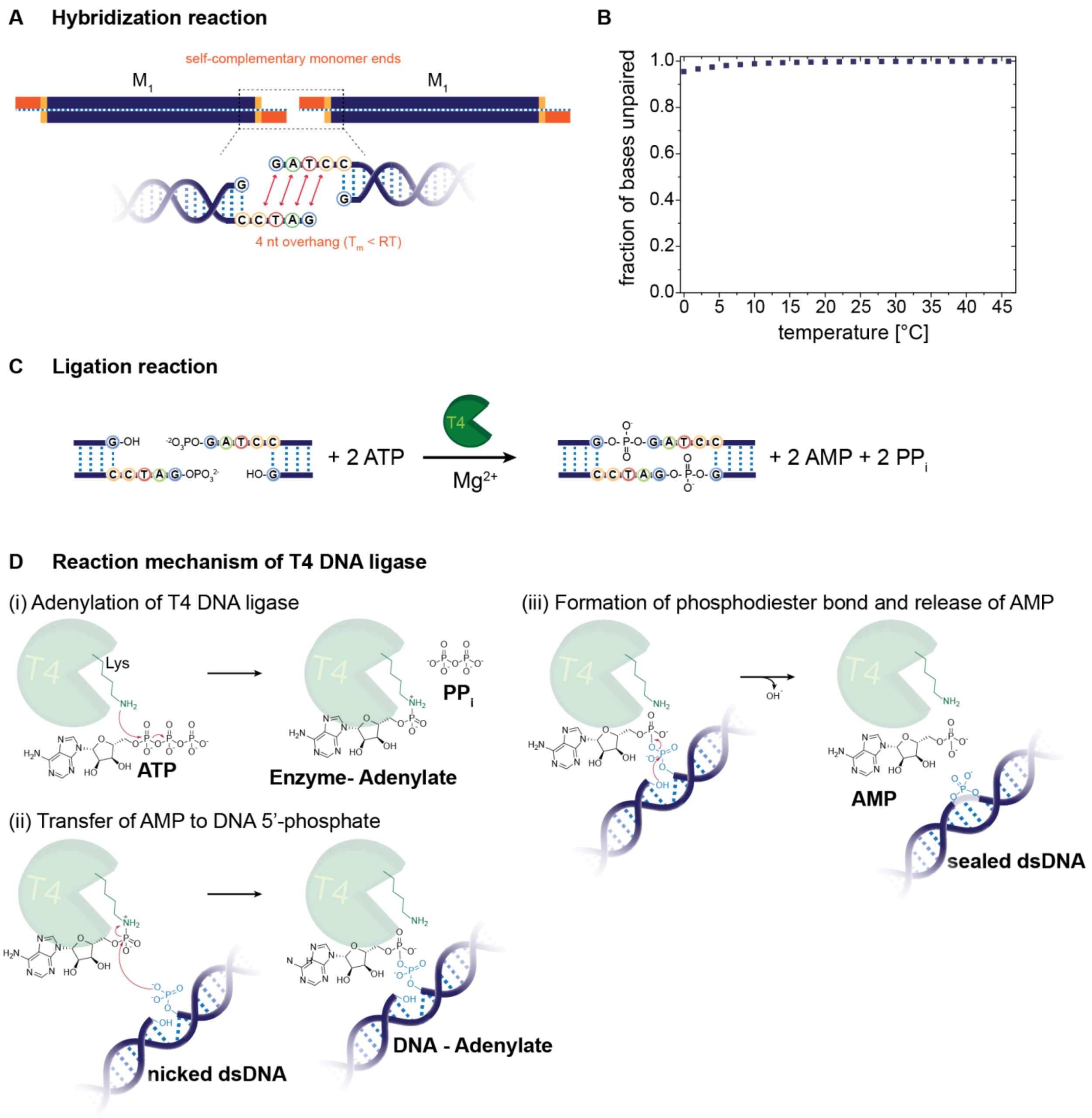

Fig. S1. Hybridization of the self-complementary ends of the DNA monomer strands M_1 in dependence of temperature and ligation reaction catalyzed by T4 DNA ligase. (A) Scheme of the self-hybridization of M_1. M_1 consists of a 34 bp duplex body with a single-stranded 4 nt overhang at each side. These ends are self-complementary, but too short to hybridize permanently with each other. (B) The predicted melting profile of this 4 bp hybridization shows that the bases are largely unpaired over the whole temperature range from 0 °C to 45 °C (conditions similar to a typical polymerization system: 0.05 mM DNA, 100 mM Na^+ and 6 mM Mg^{2+}). (C) Ligation reaction of two DNA monomer strands M_1 as seen in our step growth reaction of DNA chains. Covalent coupling of two monomer strands consumes two molecules of ATP as the formation of a phosphodiester bond in each single strand is catalyzed by an ATP-dependent T4 DNA ligase reaction. (D) Three-step reaction mechanism of T4 DNA ligase catalyzing the formation of a phosphodiester bond between the adjacent 3'-hydroxyl and 5'-phosphate group in a nicked DNA duplex.

1.2. Definition of Activity Units of both Enzymes

Definition of the Weiss Unit to describe the activity of T4 DNA ligase (Promega): 0.01 Weiss Unit [WU] of T4 DNA Ligase is the amount of enzyme required to catalyze the ligation of greater than 95% of 1 µg of λ/HindIII fragments at 16 °C in 20 minutes.
Unit definition to describe the activity of BamHI (Promega): One Unit [U] is defined as the amount of enzyme required to completely digest 1 µg of lambda DNA in one hour at 37 °C in 50 µl assay buffer containing acetylated BSA added to a final concentration of 0.1 g/L.

1.3. Ligation Kinetics of the DNA Chain Growth in Dependence of T4 DNA Ligase

The kinetics of the T4 DNA ligase-catalyzed polymerization of the M_1 monomer strands (0.05 mM) were analyzed at 25 °C. Variation of the enzyme concentration from 5.5 WU to 110 WU increases the turnover in the system, which leads to faster built-up of the polymer chains until a constant plateau is reached (fig. S2H). All ligation experiments were carried out with an excess of 1 mM ATP to exclude any conversion-related limitations of this step-growth polymerization.

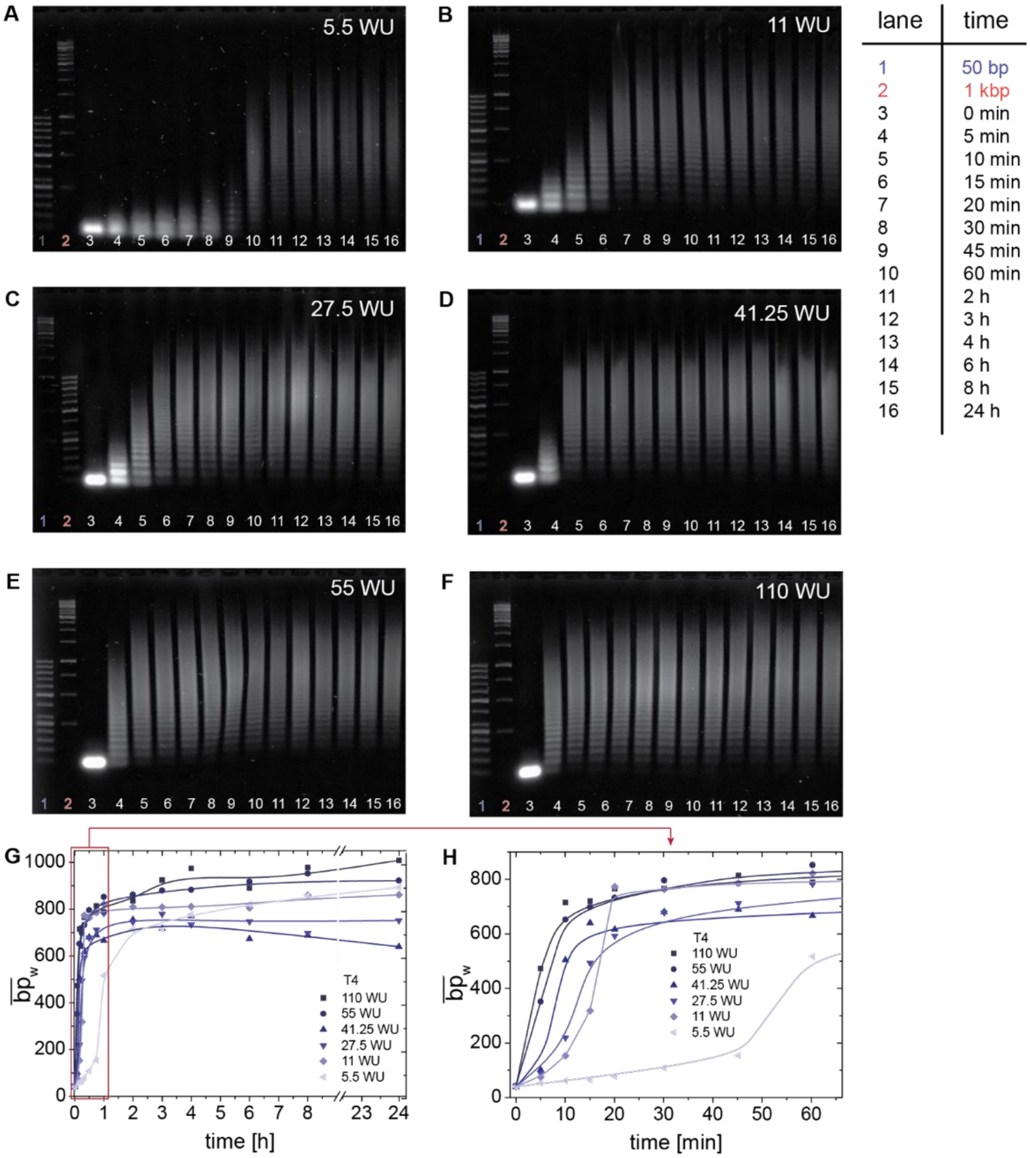

Fig. S2. Ligation kinetics of the DNA chain growth as a function of T4 DNA ligase concentration. (A to F) GE images of kinetic assays with increasing T4 DNA ligase concentration from 5.5 WU to 110 WU. (G) Evolution of the average DNA chain length $\overline{bp}_w$ with time (see Supplementary Note B for details). (H) Magnification of the initial growth phase shows acceleration with increasing T4 DNA ligase concentration. Conditions: 25 °C, 1x buffer E, 0.1 g/L BSA, 0.05 mM M_1, 1.0 mM ATP and varying T4 DNA ligase concentration. Lane assignment: 1: 50 bp ladder, 2: 1 kbp ladder, 3: 0 min, 4: 5 min, 5: 10 min, 6: 15 min, 7: 20 min, 8: 30 min, 9: 45 min, 10: 60 min, 11: 2 h, 12: 3 h, 13: 4 h, 14: 6 h, 15: 8 h, 16: 24 h.

1.4. Ligation Kinetics of the DNA Chain Growth in Dependence of ATP

Due to the step-growth character of the ATP-dependent T4 DNA ligase-catalyzed polymerization of the DNA monomer M_1, the amount of supplied ATP can be used to purposely limit the conversion, and, thus, the degree of polymerization (= average chain length) of the M_1-based DNA polymers. A substoichiometric amount of ATP compared to the theoretical number of ligation sites (= 2x monomer concentration) reduces the average chain length of the DNA polymer chains drastically (fig. S3A-D). High molecular weights can only be reached at high conversions, which require at least equimolar amounts of ATP (fig. S3E,F).

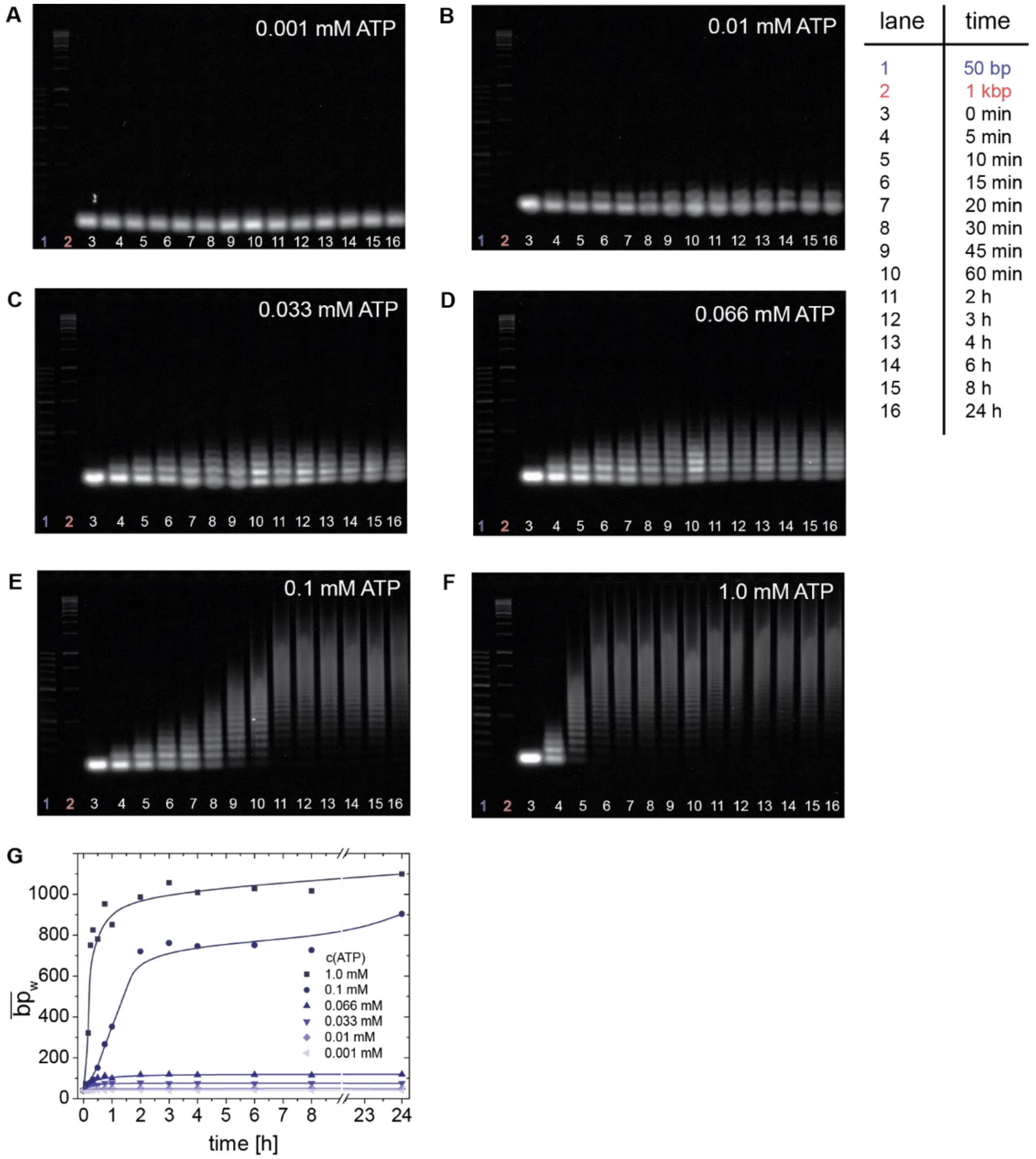

Fig. S3. Ligation kinetics of the DNA chain growth as a function of ATP concentration. (A to F) GE images of kinetic assays with increasing ATP concentration from 0.001 mM to 1.0 mM. (G) Evolution of the average DNA chain length $\overline{\boldsymbol{bp}}_{\boldsymbol{w}}$ with time. Conditions: 25 °C, 1x buffer E, 0.1 g/L BSA, 0.05 mM M_1, 41.25 WU T4 DNA ligase and varying concentrations of ATP. Lane assignment: 1: 50 bp ladder, 2: 1 kbp ladder, 3: 0 min, 4: 5 min, 5: 10 min, 6: 15 min, 7: 20 min, 8: 30 min, 9: 45 min, 10: 60 min, 11: 2 h, 12: 3 h, 13: 4h, 14: 6 h, 15: 8 h, 16: 24 h.

1.5. Long-Term Development of T4 DNA Ligase-Catalyzed DNA Chain Growth and Time-Dependent Activity Assay of the T4 DNA Ligase

The maximum molecular weight attainable for the M_1-based DNA polymers is given by the extent of the step growth reaction. A T4 DNA ligase-catalyzed polymerization reaction (standard conditions: 25 °C, 0.05 mM M_1, 41.25 WU T4 DNA ligase and excess 1 mM ATP) was carried out over a continued period of nine days to monitor how quickly maximum conversion is obtained. fig. S4A,B displays a rapid growth of the DNA chains, which already levels off after the first hours and stays constant thereafter at around $\overline{bp}_{\mathrm{w}}$ = 1200 bp. This excludes reduction of the average chain lengths due to time-limited conversion. However, given the complex nature of the system, the maximum chain lengths of the polymerization system may also be affected by slight batch-to-batch variations in reactant quality (e.g. enzyme activity, DNA purity and end group functionalization). To ensure comparability and reproducibility, all measurements within one experimental series of the kinetic studies were carried out using the same batches of reactants (DNA, enzymes, ATP).

The stability of the T4 DNA ligase over prolonged periods of time is of utmost importance for enabling the ATP-fueled transient DySS polymerization system, as it ensures the continuous energy input into the system via conversion of ATP and because it maintains the dynamic non-equilibrium steady-state character of the dynamic covalent system with continuous joining of phosphodiester bonds. The time-dependent T4 DNA ligase activity was assayed by short test ligation reactions (30 min reaction time). To this end, the DNA monomer fragment M_1 was incubated with T4 DNA ligase in a reaction tube under the standard reaction conditions at 25 °C (without ATP). Aliquots were taken on a daily basis and supplemented with fresh ATP to test for ligation efficiency. fig. S4C,D demonstrates that the obtained average chain length $\overline{bp}_{\mathrm{w}}$ of the polymer chains stays fairly constant over the investigated experimental time frame of 12 days. Minor decreases of enzyme activity do not interfere with the overall integrity of the DySS polymerization system.

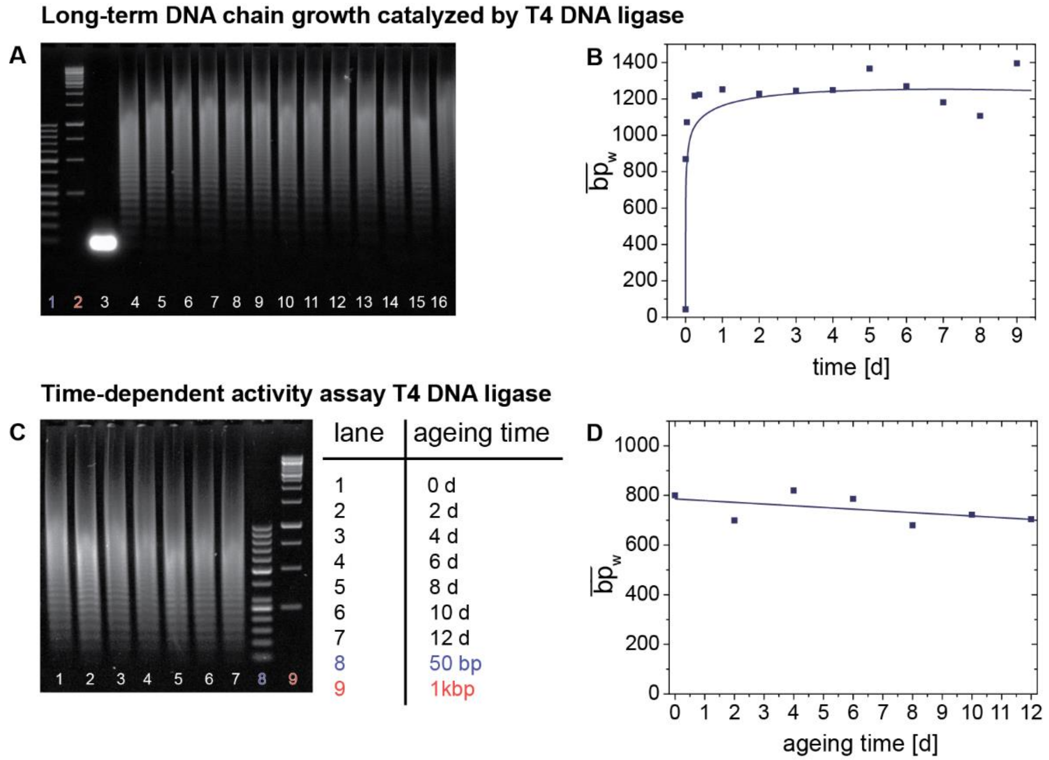

Fig. S4. Time-dependent T4 DNA ligase catalyzed ligation reaction. Long-term kinetic analysis of the T4 DNA ligase-catalyzed ligation of the DNA chain growth. (A) GE image of the time-dependent ligation assay of M_1. (B) Evolution of the average DNA chain length $\overline{bp}_w$ with time. Assay conditions: 25 °C, 1x buffer E, 0.1 g/L BSA, 0.05 mM M1, 41.25 WU T4 DNA ligase and 1.0 mM ATP. Lane assignment: 1: 50 bp ladder, 2: 1 kbp ladder, 3: 0 min, 4: 10 min, 5: 1 h, 6: 6 h, 7: 9 h, 8: 24 h, 9: 2 d, 10: 3 d, 11: 4 d, 12: 5 d, 13: 6 d, 14: 7 d, 15: 8 d, 16: 9 d. (C) Ageing of T4 DNA ligase. GE image of samples obtained after injecting ATP into T4 DNA ligase/M_1 solutions that were aged for different time frames as indicated. The polymerization time was set constant to 30 min. (D) Evolution of the average DNA chain length $\overline{bp}_w$ with time. The line is a linear fit to the data and indicates a slight loss of activity. Ligation reaction conditions: 25 °C, 1x buffer E, 0.1 g/L BSA, 0.05 mM M_1, 0.46 WU/µl T4 DNA ligase, 1.0 mM ATP and incubation for 30 min. Lane assignment: 1: 50 bp ladder, 2: 1 kbp ladder, 3: 0 min, 4: 1 d, 5: 2 d, 6: 3 d, 7: 4 d, 8: 5 d, 9: 6 d, 10: 7 d, 11: 8 d, 12: 9 d, 13: 10 d, 14: 50 bp, 15: 1 kbp.

1.6. Restriction Kinetics of the DNA Chain Degradation in Dependence of BamHI

An understanding of the time scales of the DNA cleavage in comparison to the ligation is critical to set up a suitable concentration ratio between the antagonistic enzymes, that fulfills the kinetic boundary condition for generating a transient system with faster activation than deactivation. We analyzed the restriction kinetics of BamHI, independently of the T4 DNA ligase, using previously ligated and heat-deactivated M_1-based DNA polymers P_1 as substrate. Increasing amounts of BamHI (450 U to 1800 U) strongly accelerate the digestion reaction to the original monomer M_1. The speed of the digestions scales with the BamHI concentration. Full digestion of P_1 takes up to four days for all BamHI concentrations and is hence much slower than the ligation reaction (see fig. S2). This enables the kinetic condition necessary in a non-equilibrium DySS polymerization.

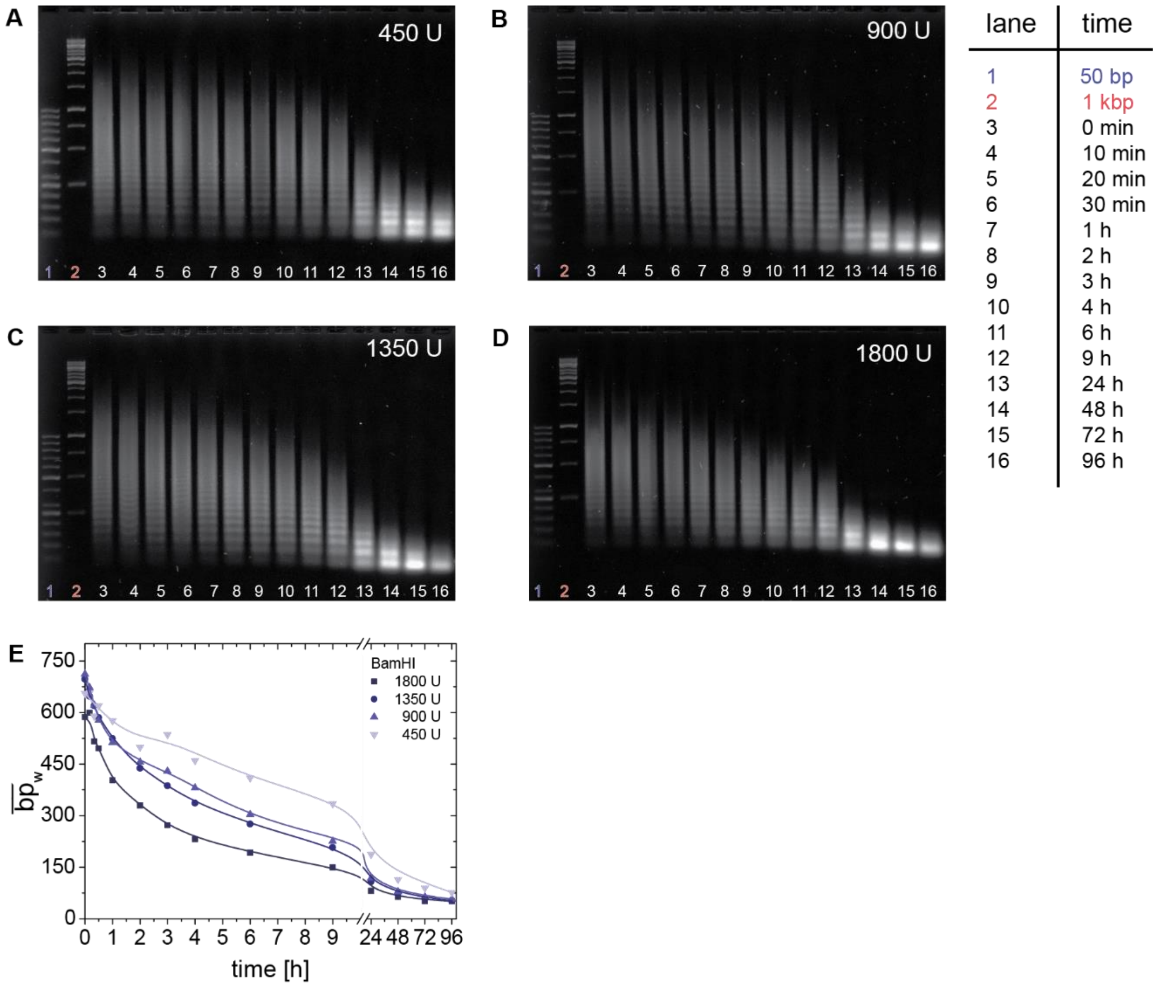

Fig. S5. Restriction kinetics of the DNA chain cleavage as a function of Bam HI concentration. (A to D) GE images of kinetic assays of the cleavage of long M_1-based DNA polymers, P_1, with increasing BamHI activity from 450 U to 1800 U. (E) Evolution of the average DNA chain length $\overline{\boldsymbol{bp}_{\boldsymbol{w}}}$ with time. Assay conditions: 25 °C, 1x buffer E, 0.1 g/L BSA, 0.05 mM P1 (polymer of M_1), and varying concentrations of BamHI. Lane assignment: 1: 50 bp ladder, 2: 1 kbp ladder, 3: 0 min, 4: 10 min, 5: 20 min, 6: 30 min, 7: 1 h, 8: 2 h, 9: 3 h, 10: 4 h, 11: 6 h, 12: 9 h, 13: 24 h, 14: 48 h, 15: 72 h, 16: 96 h.

Supplementary Note B. Routine of GE analysis: From the agarose gel to an average chain length

The enzyme-catalyzed step growth reaction of the DNA monomer, M_1, leads to a distribution of DNA chains with a broad range of molecular weights (38 bp to > 10000 bp). GE allows separation of DNA chains with molecular resolution down to a few base pairs (bp). Band resolution scales mainly with the agarose concentration (i.e. effective pore size of the gel), applied voltage and run time. GE conditions were optimized in TAE buffer to satisfy imaging of the broad chain length distribution. We chose an agarose concentration of 2 wt% (90 V, 90 min) to identify clearly the number of single repeat units of the oligomers (monomer, dimer, trimer, …) as illustrated in fig. S6A.

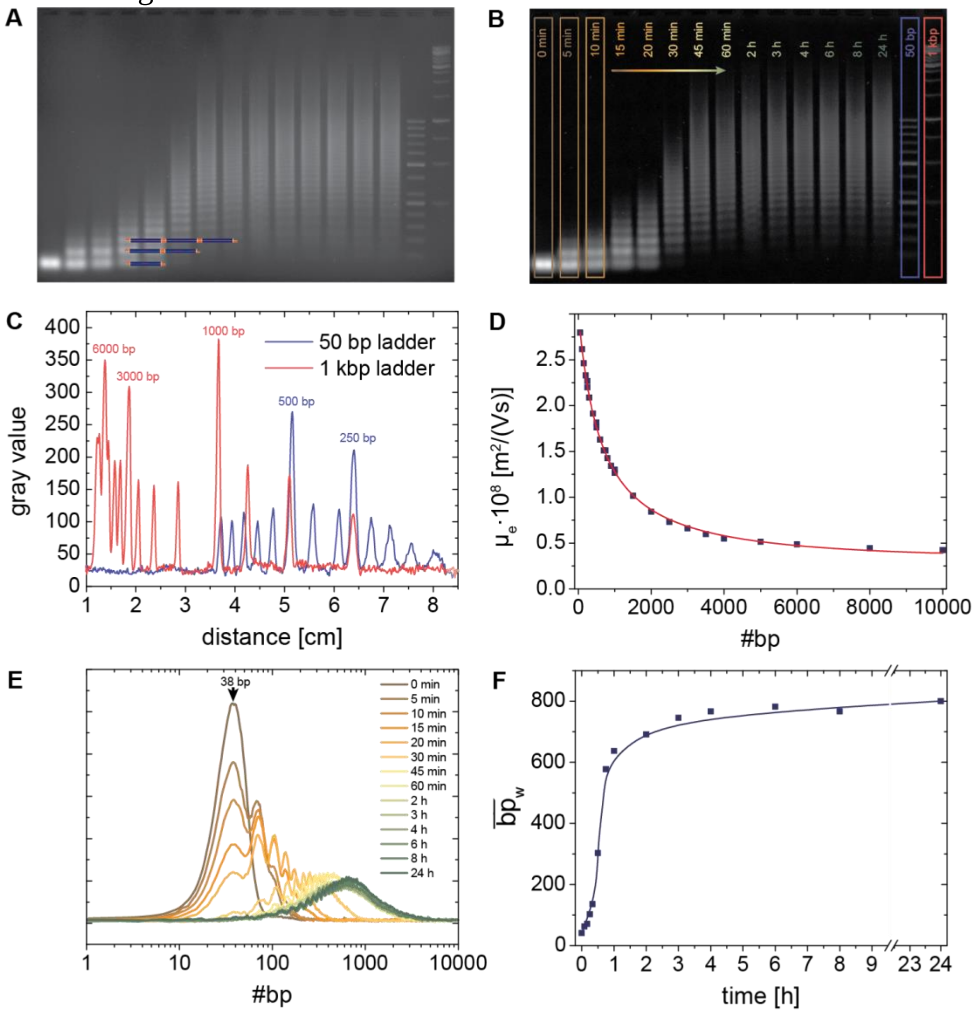

Fig. S6. Routine for analysis of GE data: From the agarose GE to an average DNA chain length $\overline{bp}_w$. (A) Raw GE image of a time-dependent ligation experiment. The DNA base pair ladders (50 bp and 1 kbp) used for calibration are on the right-hand side. (B) Background-corrected GE image. (C) Extracted gray scale profiles of the base pair ladders. (D) Calibration curve to convert electrophoretic mobility into number of base pairs (#bp) based on the band assignment of the DNA ladders. (E) Time-dependent gray scale profiles of the ligation assay as a function of #bp aligned to the position of the 38 bp band of M_1. (F) Evolution of the average DNA chain length ($\overline{bp}_w$) with time.

Band resolution gets poorer, i.e. molecular weight separation is less resolved, for longer DNA chains, which effectively cuts down the tail of the molecular weight distribution and reduces the measured average chain length and dispersity. DNA staining is based on Roti-GelStain, a benzimidazole dye binding to the minor groove of the DNA double helix. The binding scales with the length of the DNA duplex. Thus, the fluorescence signal obtained from the DNA staining is proportional to the DNA concentration and chain length (its mass), and the extracted gray scale profile from each lane of the agarose GE images corresponds to a mass-weighted chain length distribution.

DNA base pair ladders allow a calibration of the GE images and calculation of an average chain length as demonstrated below. The GE images of the kinetic electrophoretic mobility shift assays were routinely analyzed to obtain quantitative data on the development of the average chain length over time. The procedure is shown in fig. S6 using the example of a time-dependent ligation reaction of the DNA chain growth. First, the raw GE image (fig. S6A) is background corrected (fig. S6B) using the sliding paraboloid algorithm to remove uneven illumination (Image J 1.51k). All GE images shown in the manuscript/supporting materials are background-corrected. Afterwards gray scale profiles of each lane are extracted, giving intensity values as a function of migrated distance (fig. S6 C). The migrated distances d_m are converted into electrophoretic mobilities μ_e according to Eq. 1 with drift velocity v, electric field strength E, time t, voltage V, distance between the electrodes d_el.

$$\mu_\mathrm{e} = \frac{v}{E} = \frac{\frac{d_\mathrm{m}}{t}}{\frac{V}{d_\mathrm{el}}} \qquad \text{Equation 1}$$

A 50 bp and 1 kbp DNA ladder are used for calibration of the gels by fitting electrophoretic mobility μ_e as a function of DNA chain length x [bp] as described by Van Winkle et al.(*41*) in the following relationship (Eq. 2) with α, β, and γ as adjustable fit parameters (fig. S6C, D).

$$\mu_\mathrm{e}(x) = \frac{1}{\beta + \alpha\left(1 - e^{-\frac{x}{\gamma}}\right)} \qquad \text{Equation 2}$$

This fit function is used to recalculate the gray scale profiles as a function of base pair number. The band position of the monomer strand is normalized to its length of 38 bp (fig. S6E). From this chain length distribution, the mass average chain length $\overline{bp_w}$ is obtained via applying Eq. 3 with bp_i as the base pair number of fraction i and f_i as the intensity value of fluorescence intensity of the corresponding fraction i.

$$\overline{bp_w} = \frac{\sum_{\mathrm{i}=1}^{\mathrm{n}} f_\mathrm{i} \cdot bp_\mathrm{i}}{\sum_{\mathrm{i}=1}^{\mathrm{n}} f_\mathrm{i}} \qquad \text{Equation 3}$$

The average DNA chain length $\overline{bp_w}$ is plotted over time to estimate the temporal evolution of the enzymatic polymerization reaction (fig. S6F).

Supplementary Note C. ATP-fueled transient, dynamic steady-state DNA polymerization system

1.7. Control of Dispersity in the DySS DNA Polymerization System

In principal, the DySS DNA polymerization system is a catalytically controlled and energy-driven step growth polymerization, which follows Carother's equation ($X_n = 1/(1-p)$). The dispersity is a function of the conversion according to $Đ = M_w/M_n = 1+p$, where p is the conversion. Since the conversion is an adaptive function of the effective enzyme ratio, one could describe the dispersity as such. The dispersity can be calculated, as the GE data provides the whole data set of a complete molecular weight distribution. The pure ligation reaction (static polymerization) follows very well Carother's prediction and gives $Đ = 2$ for full conversion ($p = 1$, ca. 100% ligation = limiting case, fig. S7A). The dispersity of the ATP-fueled dynamic polymerization system depends on the DySS conversion which is given by the balanced, effective ligation and cleavage rates, i.e. primarily determined by the enzyme ratio and the temperature as shown below in fig. S7B for the dependence of the DySS polymerization on the temperature. The DySS dispersity data in fig. S7B corresponds to the experiments shown in Fig. 3E in the manuscript.

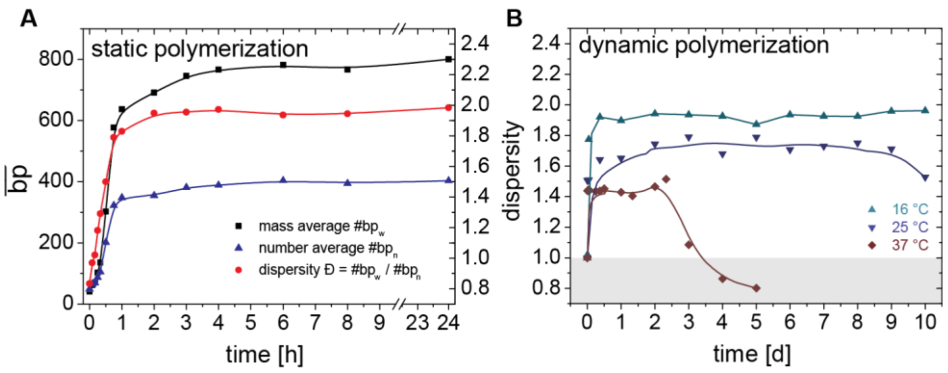

Fig. S7. Control of dispersity in the DySS DNA polymerization system. (A) Time-dependent evolution of the dispersity $Đ$ in a *static* polymerization of M_1 catalyzed by T4 DNA ligase and excess of ATP. Eventually, $Đ$ approaches 2 in agreement with a step growth polymerization reaction following Carother's equation of $Đ = 1 + p$. Kinetic assay conditions: 25 °C, 1x buffer E, 0.1 g/L BSA, 41.25 WU T4 DNA ligase, 1.0 mM ATP and 0.1 mM M_1. (B) Dispersity $Đ$ over time in a transient DySS polymerization of M_1 controlled by T4 DNA ligase and BamHI with a DySS conversion in dependence of temperature. See Figure 3E in the main manuscript. Kinetic assay conditions: 1x buffer E, 0.1 g/L BSA, 41.25 WU T4 DNA ligase, 900 U BamHI, 1.0 mM ATP and 0.05 mM M_1 at 16 °C, 25 °C and 37 °C. Dispersity $Đ < 1$ are physically invalid and hint at the limitations during the evaluation of GE data.

1.8. Refueling Experiments of the Transient DySS DNA Polymerization System

The aim of the refueling experiments is to clearly identify ATP as the driver of a chemically fueled dynamic polymerization reaction. To this end, a standard DySS polymerization reaction was initiated with 0.1 mM of ATP (fig. S8). After completion of the first cycle, the system was supplemented with fresh reactants: A) ATP, B) T4 DNA ligase, C) ATP and T4 DNA ligase, and

D) no reagent (negative control). A second transient polymerization cycle is only observed for the experiments that include further addition of ATP. Critically, the addition of fresh T4 (B) does not reinitiate the polymerization, proving that no loss of T4 DNA ligase enzyme activity causes the transient character of the polymerization. A similar behavior is obtained for the same refueling and control experiments at 37 °C, underscoring the robustness at higher temperature (Manuscript Fig. 3). Refueling experiments monitored by FRET are presented in fig. S11.

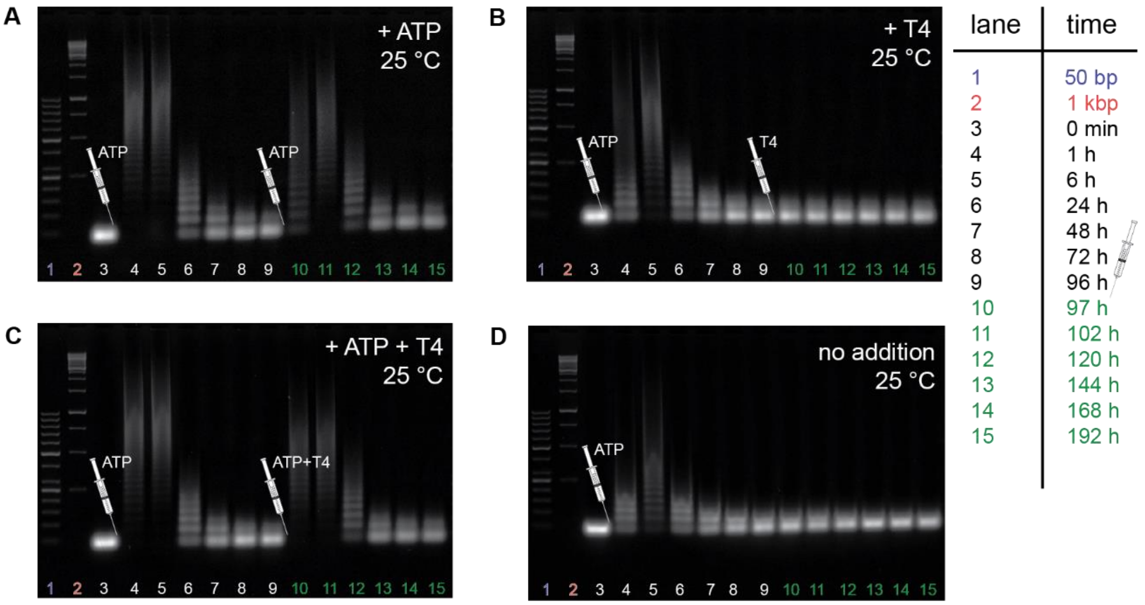

Fig. S8. Refueling experiments of the transient DySS DNA polymerization system. After completion of the first lifetime cycle (96 h), the systems are injected with (A) ATP (0.1 mM), (B) T4 DNA ligase (0.46 WU/µl), (C) ATP (0.1 mM) and T4 DNA ligase (0.46 WU/µl), (D) no reagents added. The concentrations of the reactants added during the second injection are equivalent to those applied in the first lifetime cycle. The GE images of the kinetic assays show successful re-initiation of the transient DySS DNA polymerization only in conjunction with ATP (A, C). Kinetic assay conditions: 25 °C, 1x buffer E, 0.1 g/L BSA, 0.05 mM M_1, 41.25 WU T4 DNA ligase (0.46 WU/µl), 900 U BamHI (10 U/µl) and 0.1 mM ATP. Lane assignment: 1: 50 bp ladder, 2: 1 kbp ladder, 3: 0 min, 4: 1 h, 5: 6 h, 6: 24 h, 7: 48 h, 8: 72 h, 9: 96 h, 10: 97 h, 11: 102 h, 12: 120 h, 13: 144 h, 14: 168 h, 15: 192 h.

1.9. Transient DySS DNA Polymerization in Dependence of the DNA Concentration

The concentration of the DNA monomer M_1 impacts drastically the temporal evolution of the transient enzyme-catalyzed DySS polymerization with respect to the timescales needed to enter and leave the DySS and regarding the achievable average chain length of the dynamic DNA polymers. At a constant enzyme concentration ratio (41.25 WU T4 DNA ligase and 900 U BamHI) and ATP concentration (0.1 mM), higher monomer concentrations (M_1) slow down the absolute time scales of formation and degradation of the polymers as the substrate to enzyme ratio is higher, which delays high conversions needed for high molecular weights in step growth reactions and for complete digestion. Further, the different stoichiometry between DNA monomer M_1 and ATP can alter the degree of polymerization due to limitations of the chemical fuel for maximum conversion. This needs to be considered for the concentration of 0.1 mM DNA.

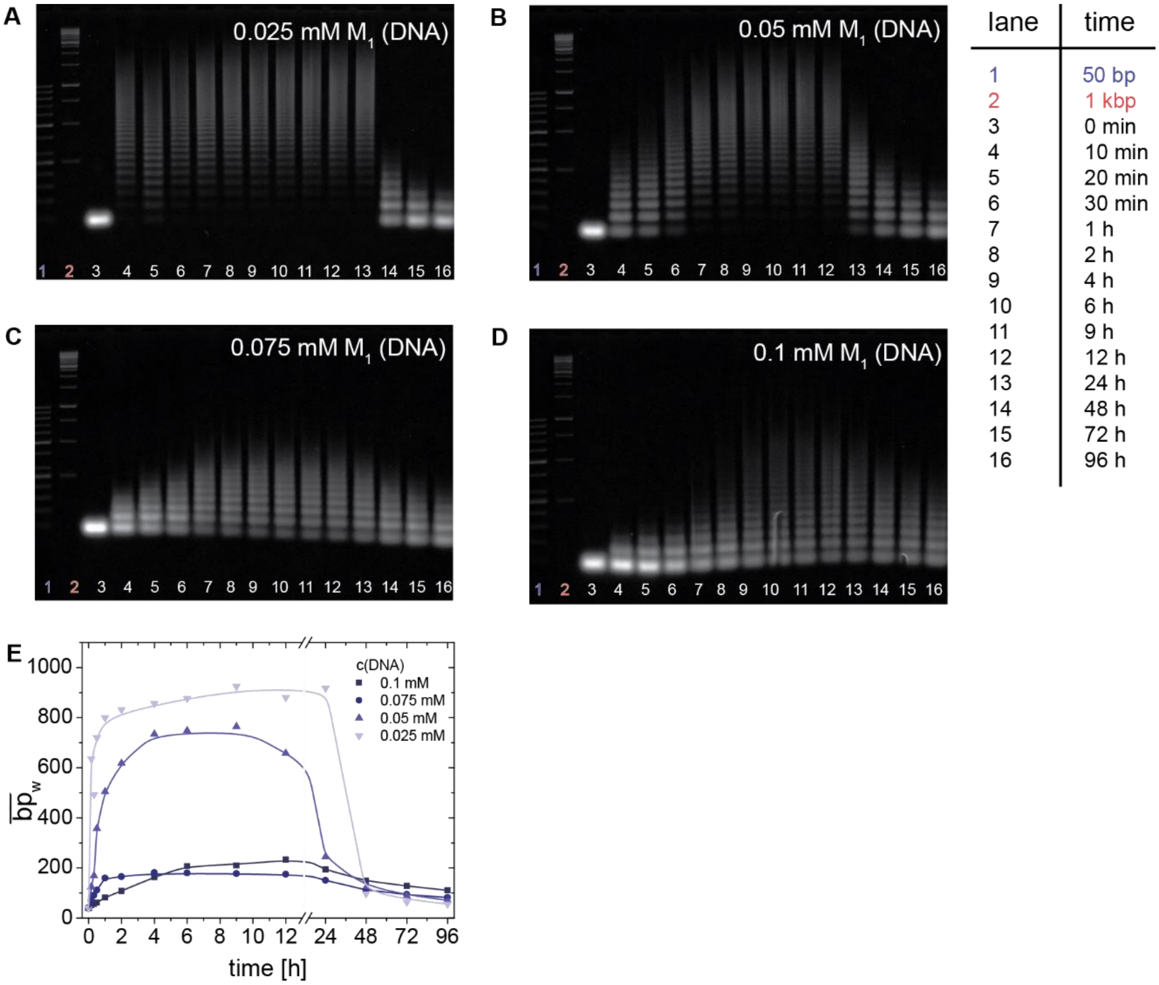

Fig. S9. Average chain length in the transient DySS DNA polymerization system in dependence of the concentration of the DNA monomer M_1. (A to D) GE images of kinetic assays with increasing DNA concentration (M_1) from 0.025 mM to 0.1 mM. (E) Evolution of the average DNA chain length $\overline{bp}_w$ with time. Kinetic assay conditions: 25 °C, 1x buffer E, 0.1 g/L BSA, 41.25 WU T4 DNA ligase, 900 U BamHI, 0.1 mM ATP and varying concentrations of M_1. Lane assignment: 1: 50 bp ladder, 2: 1 kbp ladder, 3: 0 min, 4: 10 min, 5: 20 min, 6: 30 min, 7: 1 h, 8: 2 h, 9: 4 h, 10: 6 h, 11: 9 h, 12: 12 h, 13: 24 h, 14: 48 h, 15: 72 h, 16: 96 h.

Supplementary Note D. DySS and molecular exchange in ATP-fueled dissociative dynamic covalent DNA systems

1.10. Characterization of the Different States of the FRET Duplex F

The FRET duplex F (42 bp) carries the FRET pair Cy3/Cy5 as fluorescent tags around the BamHI restriction site which is located at the center of the sequence. The FRET pair serves as a reporter for the extent of the ensemble average steady-state covalent bonding in the dynamized phosphodiester bond. The dyes are attached via amino-C6 linkers to thymines in the complementary strands 10 bp apart from each other. The fluorescent tags show no adverse effects on the enzymatic cleavage and religation as shown by the clean reactions (restriction, religation) in GE (fig. S10A).

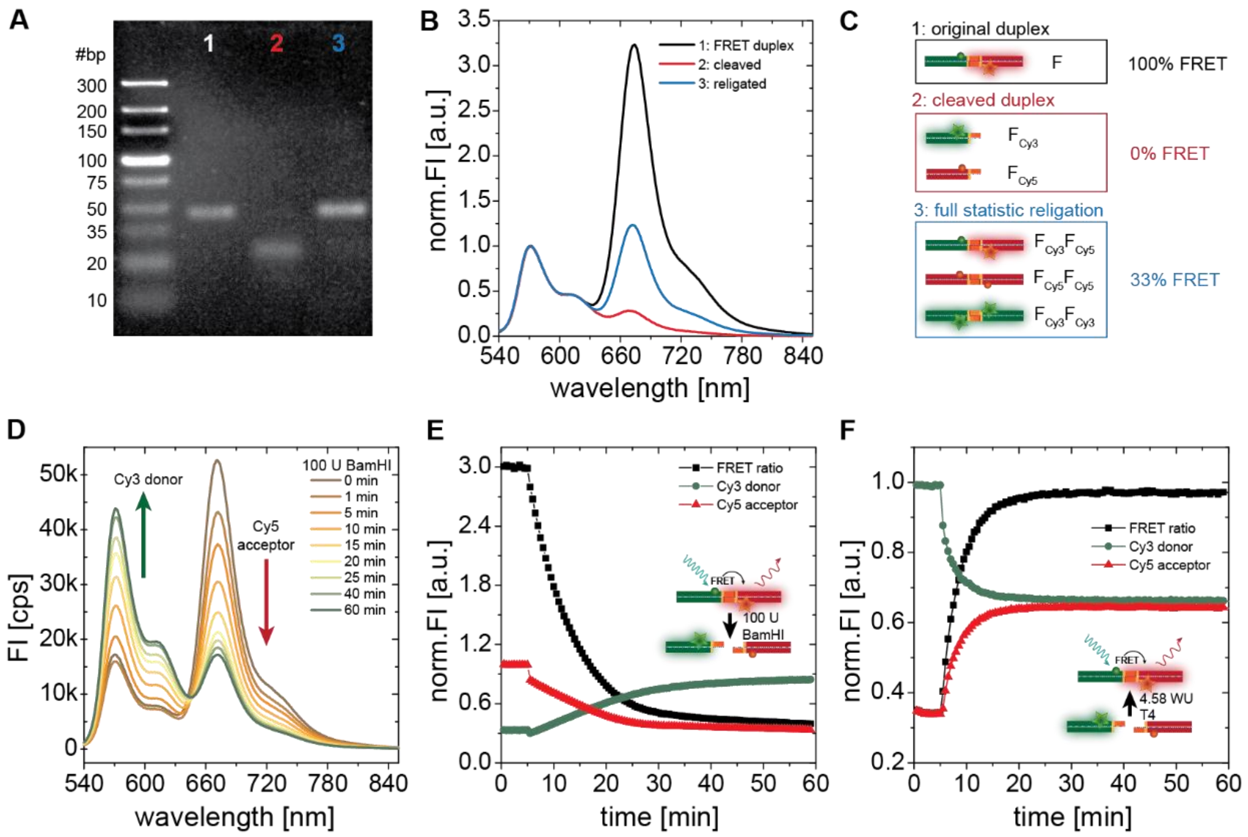

Fig. S10. Characterization of the FRET duplex F and its cleaved and religated DNA fragments as used for in situ modulation of the DySS. (A) The FRET duplex F with the fluorescent tags Cy3 and Cy5 next to the restriction/ligation site can be cleaved by BamHI and religated by T4 DNA ligase completely without any residual traces as seen by the single DNA bands in GE. Lane assignment: 1: original FRET duplex, 2: cleaved state, 3: religated state. (B) Corresponding fluorescence spectra normalized to the Cy3 donor emission peak at 571 nm. The religated FRET duplexes show 1/3 of the original FRET emission at 674 nm (Cy5 acceptor) due to statistic recombination of the cleaved DNA duplex fragments F_{Cy3} and F_{Cy5} to $F_{Cy3}F_{Cy3}$, $F_{Cy5}F_{Cy5}$ and $F_{Cy3}F_{Cy5}$ (1:1:1). Critically, only $F_{Cy3}F_{Cy5}$ induces FRET. (C) Schematic representation of the different FRET duplex bonding states. (D) Fluorescence spectra during cleavage of the original FRET duplex F with 100 U BamHI, and (E) evaluation of the Cy3 donor emission at 571 nm (green dot), the Cy5 acceptor emission at 674 nm (red triangle) and the FRET ratio (674 nm/571 nm, black squares) over time. (F) Religation kinetics of the cleaved FRET duplex fragments (F_{Cy3}, F_{Cy5}) with 4.58 WU and 4 µM ATP as represented by the time-dependent fluorescence signals of the Cy3 donor at

571 nm (green dot), the Cy5 acceptor at 674 nm (red triangle) and the FRET ratio (674 nm/571 nm, black squares). All fluorescence spectra were recorded in 1x buffer E with 1 µM F, 0.2 g/L BSA, $\lambda_{exc.}$ = 505 nm, 1 s integration time, 25 °C.

The original FRET duplex F hybridized from strand F_a and F_b shows a maximum FRET emission at 674 nm (= 100 %, black line, fig. S10B), because all Cy3 donors are positioned next to their Cy5 acceptor FRET partners. After complete enzymatic cleavage with BamHI, both dyes are fully separated into two duplex fragments (F_{Cy3} and F_{Cy5}, both 21 bp in length) with no FRET being observed (= 0 %, red line, fig. S10B). Upon religation of these cleaved duplex fragments by T4 DNA ligase the maximum FRET emission does not and cannot recover to the original value. This is due to a statistic recombination of the palindromic (self-complimentary) ends of the cleaved parts. Religation of the cleaved DNA duplex fragments F_{Cy3} and F_{Cy5} leads to $F_{Cy3}F_{Cy3}$, $F_{Cy5}F_{Cy5}$ and $F_{Cy3}F_{Cy5}$ in a 1:1:1 ratio. Critically, only $F_{Cy3}F_{Cy5}$ shows FRET. The possible reaction products upon religation are illustrated in fig. S10C. Hence, the FRET efficiency is reduced to 1/3 (= 33 %, blue line) of the original state. Ultimately, the fully cleaved and religated state represent the limiting cases of the dynamic DNA bond formation, and, thus, the accessible and tunable range of the fluorescence spectra changes due to FRET in the DySS experiments presented in the manuscript (Figure 4) and Section 1.11.

Figure S10D-F show the spectral changes during cleavage of F and of the religation of F_{Cy3} and F_{Cy5}. Enzymatic cleavage of the FRET duplex reduces the FRET-induced emission of the Cy5 acceptor at 674 nm and strengthens the Cy3 donor emission at 571 nm (fig. S10D,E). The opposite behavior is observed for the ligation reaction on the cleaved DNA by reformation of the FRET pair (however to a lesser extent due to statistical intermixing). In the following, the FRET ratio (Cy5/Cy3 = $I_{674\ nm}/I_{571\ nm}$) is used for the time-resolved evaluation of the dynamic bonding state of the FRET duplex and converted to a relative percentage of ligation using the accessible FRET signal limits of the DySS. Ultimately, this relative degree of bonding translates into an ensemble average steady-state bond strength of the dynamic system. We observed that the presence of the DNA-bound enzymes slightly lowers the FRET efficiency between the Cy3/Cy5 pair.

1.11. ATP-Dependent Temporal Control of the Dynamic DNA Bond Visualized by Time-Dependent FRET

The average lifetime of the dynamic covalent DNA bond, as represented here by the dynamic transient ligation of the FRET duplex fragments, F_{Cy3} and F_{Cy5}, can be controlled by the amount of ATP as chemical fuel. Increasing amounts of ATP elongate the DySS phase by several hours under the chosen conditions (fig. S11A,B). Transient dynamic ligation can be reactivated multiple times when supplemented with new ATP (fig. S11C).

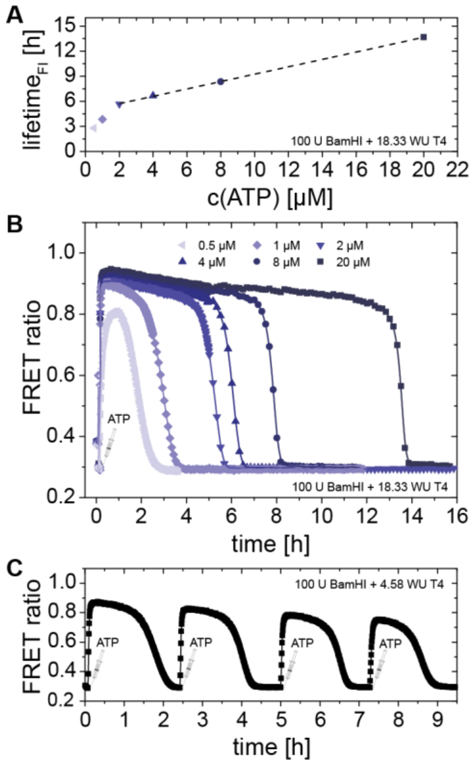

Fig. S11. ATP-dependent temporal control of the dynamic DNA bond with transient DySS FRET duplex formation. Programming of the transient lifetime of the dynamic FRET duplex (1 µM cleaved F (F_{Cy3} + F_{Cy5})) is shown for an enzyme ratio of 18.33 WU T4 DNA ligase and 100 U BamHI with varying amounts of ATP as chemical fuel in 1x buffer E with 0.2 g/L BSA at 25 °C. (A) Evaluated lifetime and (B) corresponding time-dependent traces of the FRET ratio for ATP concentrations from 0.5 µM to 20 µM. Molar ratios of ATP to ligation sites > 1 suggest a linear dependency of the lifetime on the ATP concentration (dashed line). This is in line with the trend presented in Figure 2 (manuscript). (C) After completion of one full transient cycle, the transient DySS cycle can be reinitiated multiple times by addition of ATP. The refueling experiment shows four consecutive cycles each initiated with 2 µM ATP in a dynamic system of 100 U BamHI and 4.58 WU T4 DNA ligase with 1 µM cleaved F (F_{Cy3} and F_{Cy5}), 0.2 g/L BSA in 1x buffer E at 25 °C.
